# Exploiting innate immunity for cancer immunotherapy

**DOI:** 10.1186/s12943-023-01885-w

**Published:** 2023-11-27

**Authors:** Ming Yi, Tianye Li, Mengke Niu, Qi Mei, Bin Zhao, Qian Chu, Zhijun Dai, Kongming Wu

**Affiliations:** 1 Cancer Center, Shanxi Bethune Hospital, Shanxi Academy of Medical Science, Tongji Shanxi Hospital, Third Hospital of Shanxi Medical University, Taiyuan, 030032 People’s Republic of China; 2 Department of Breast Surgery, College of Medicine, The First Affiliated Hospital, Zhejiang University, Hangzhou, 310000 People’s Republic of China; 3 Department of Gynecology, The Second Affiliated Hospital of Zhejiang University School of Medicine, Hangzhou, 310000 People’s Republic of China; 4 Department of Oncology, Tongji Hospital of Tongji Medical College, Huazhong University of Science and Technology, 1095 Jiefang Avenue, Wuhan, 430030 People’s Republic of China

**Keywords:** Cancer immunotherapy, Innate immunity, Dendritic cell, Macrophage, Neutrophil, Natural killer cell, Myeloid-derived suppressor cell, Chimeric antigen receptor

## Abstract

Immunotherapies have revolutionized the treatment paradigms of various types of cancers. However, most of these immunomodulatory strategies focus on harnessing adaptive immunity, mainly by inhibiting immunosuppressive signaling with immune checkpoint blockade, or enhancing immunostimulatory signaling with bispecific T cell engager and chimeric antigen receptor (CAR)-T cell. Although these agents have already achieved great success, only a tiny percentage of patients could benefit from immunotherapies. Actually, immunotherapy efficacy is determined by multiple components in the tumor microenvironment beyond adaptive immunity. Cells from the innate arm of the immune system, such as macrophages, dendritic cells, myeloid-derived suppressor cells, neutrophils, natural killer cells, and unconventional T cells, also participate in cancer immune evasion and surveillance. Considering that the innate arm is the cornerstone of the antitumor immune response, utilizing innate immunity provides potential therapeutic options for cancer control. Up to now, strategies exploiting innate immunity, such as agonists of stimulator of interferon genes, CAR-macrophage or -natural killer cell therapies, metabolic regulators, and novel immune checkpoint blockade, have exhibited potent antitumor activities in preclinical and clinical studies. Here, we summarize the latest insights into the potential roles of innate cells in antitumor immunity and discuss the advances in innate arm-targeted therapeutic strategies.

## Background

During cancer evolution, accumulating point mutations and structural alterations drive malignant transformation and contribute to the immunogenicity of cancer cells [1]. Tumor antigens expressed by mutated genes could be recognized by host immunity as non-self and initiate immune elimination [2]. In immune-mediated elimination, innate immunity cooperates with adaptive immunity to orchestrate a cascade multi-step process, which begins with tumor antigen capture and ends with immune killing [3–6]. Innate immunity serves as the first front line of host defense, consisting of physical and chemical barriers and various types of immune cells with pattern-recognition receptors (PRRs). Innate immune components, involving dendritic cells (DCs), macrophages, monocytes, neutrophils, eosinophils, basophils, mast cells, natural killer (NK) cells, natural killer T (NKT) cells, γδ T cells, mucosa-associated invariant T (MAIT) cells, retard tumor growth mainly by nonspecifically killing malignant cells or mobilizing adaptive immune response [7]. In contrast with the innate arm, the adaptive arm of host immunity specifically eradicates cancer cells by T and B cells [8].

Ideally, all transformed cells are recognized and eliminated by host immunity. However, cancer is a heterogeneous disease, and a large scale of genetic and epigenetic alterations are unevenly distributed in several parallel subclones [9–11]. Under the selective pressure of adaptive immunity, tumor subclones with weak immunogenicity become the predominant subclones that escape immune-mediated tumor clearance [12]. The poor immunogenicity, coupled with multiple immunosuppressive factors such as immune checkpoint pathways, metabolite reprogramming, and dysregulated cytokine repertoire, support selected subclones to develop into clinically apparent lesions [13–18]. Besides, immunosuppressive cell populations in the tumor microenvironment (TME), including tumor-associated macrophages (TAMs), regulatory T (Treg) cells, regulatory B (Breg) cells, myeloid-derived suppressor cells (MDSCs), tumor-associated neutrophils (TANs), and cancer-associated fibroblasts (CAFs), also promote immune evasion and cancer progression [19–23].

Antitumor immunotherapies, including immune checkpoint blockade [24] and adoptive cell transfer [25–27], have been widely validated and clinically approved for various cancers. These strategies aim to eradicate cancer cells by enabling T cell-mediated antitumor responses. Immune checkpoint molecules are commonly upregulated in the TME, which hamper T cell activation by counteracting T cell receptor (TCR) signaling or attenuating the costimulatory pathway [28–30]. Immune checkpoint antibodies disturb immunosuppressive pathways in T cells, especially programmed cell death protein 1 (PD-1)-programmed cell death ligand 1 (PD-L1) and cytotoxic T lymphocyte-associated protein 4 (CTLA-4)-CD80/CD86 signaling [31, 32]. Up to now, more than ten anti-PD-1/PD-L1 antibodies have been approved for cancer treatment.

Meanwhile, adoptive cell transfer strategies, mainly chimeric antigen receptor (CAR)-T cell therapy, make a breakthrough in hematological malignancies [33, 34]. CAR-T cells are prepared by transducing genetically engineered receptors into autologous T cells [35]. These engineered TCRs contain extracellular domains recognizing tumor antigens and intracellular domains mimicking TCR activation signaling [36, 37]. At the present stage, six CAR-T cell products have been clinically approved: Yescarta (anti-CD19), Kymriah (anti-CD19), Tecartus (anti-CD19), Breyanzi (anti-CD19), Abecma (anti-BCMA), and Carvykti (anti-BCMA) for B cell malignancies and multiple myeloma [38–41]. Also, DC-targeted adoptive cell transfer strategies have made substantial headway. Provenge, autologous DC loaded with the fusion protein of granulocyte–macrophage colony-stimulating factor and prostatic acid phosphatase, has been approved for prostate cancer [42].

Although these immunotherapies have achieved tremendous success in advanced cancers, some thorny issues remain to be resolved, including the unsatisfactory response rate and lack of accurate predictors. It was estimated that 43.63% of all cancer patients were eligible for immune checkpoint blockade, and the overall response rate was below 13% in the US [43]. Besides, CAR-T cell therapy indications are limited to hematologic malignancies, without significant antitumor activity in solid tumors [44–47]. Generally, most clinically approved immunotherapies are T cell-centered. However, the effector functions of T cells are non-autonomous. The initiation and sustainability of T cell response and the maintenance of T cell memory depend on innate immunity [48]. Innate immunity detects, captures, and processes cancer antigens and then triggers adaptive immunity. At the same time, innate immune cells directly eradicate tumors by mounting their effector responses, such as the cytotoxicity of NK cells and the phagocytosis of macrophages [48]. Besides, due to the expression of Fc receptor (FcR) on macrophages and NK cells, innate immunity could participate in adaptive immunity by launching antibody-dependent cell cytotoxicity and phagocytosis (ADCC and ADCP) [49]. As the essential role of the innate immune arm in the onset, propagation, and maintenance of the cancer-immunity cycle, it is rationale to harness innate response to improve the current immunotherapy performance and relieve treatment resistance. In this work, we review the roles of innate immune components in antitumor immunity and summarize the advances in innate immunity-targeted immunotherapies.

## The role of DC in antitumor response and DC-targeted therapy

DCs are a heterogeneous group of myeloid-derived populations. According to the developmental origin, DCs are commonly classified into several subsets: conventional DC (including cDC1 and cDC2), plasmacytoid DC (pDC), monocyte-derived DC (MoDC), and tumor-infiltrating DC3 [50]. Among these subsets, cDC1 is functionally specialized in the cross-presentation of cancer antigens [51, 52], while pDC is the specialized producer of IFN-I [53]. Besides, based on tissue-specific compartmentalization, DCs could be classified as migratory DC (migDC, trafficking from peripheral tissues to draining lymph nodes) and resident DC (resDC, residing in peripheral lymphoid organs). Notably, the omics technique, especially single-cell RNA sequencing, provides a high-resolution landscape of DC differentiation and ontogeny [54]. To trigger and maintain robust antitumor response, DCs orchestrate a cascade of events: antigen capture and process, trafficking to tumor-associated draining lymph nodes (tdLNs), priming naïve T cells, recruiting primed T cells into the TME by secreting chemokines, and interacting with effector T cells in the TME [55].

### Innate sensing and cancer antigen presentation

**Fig. 1:**
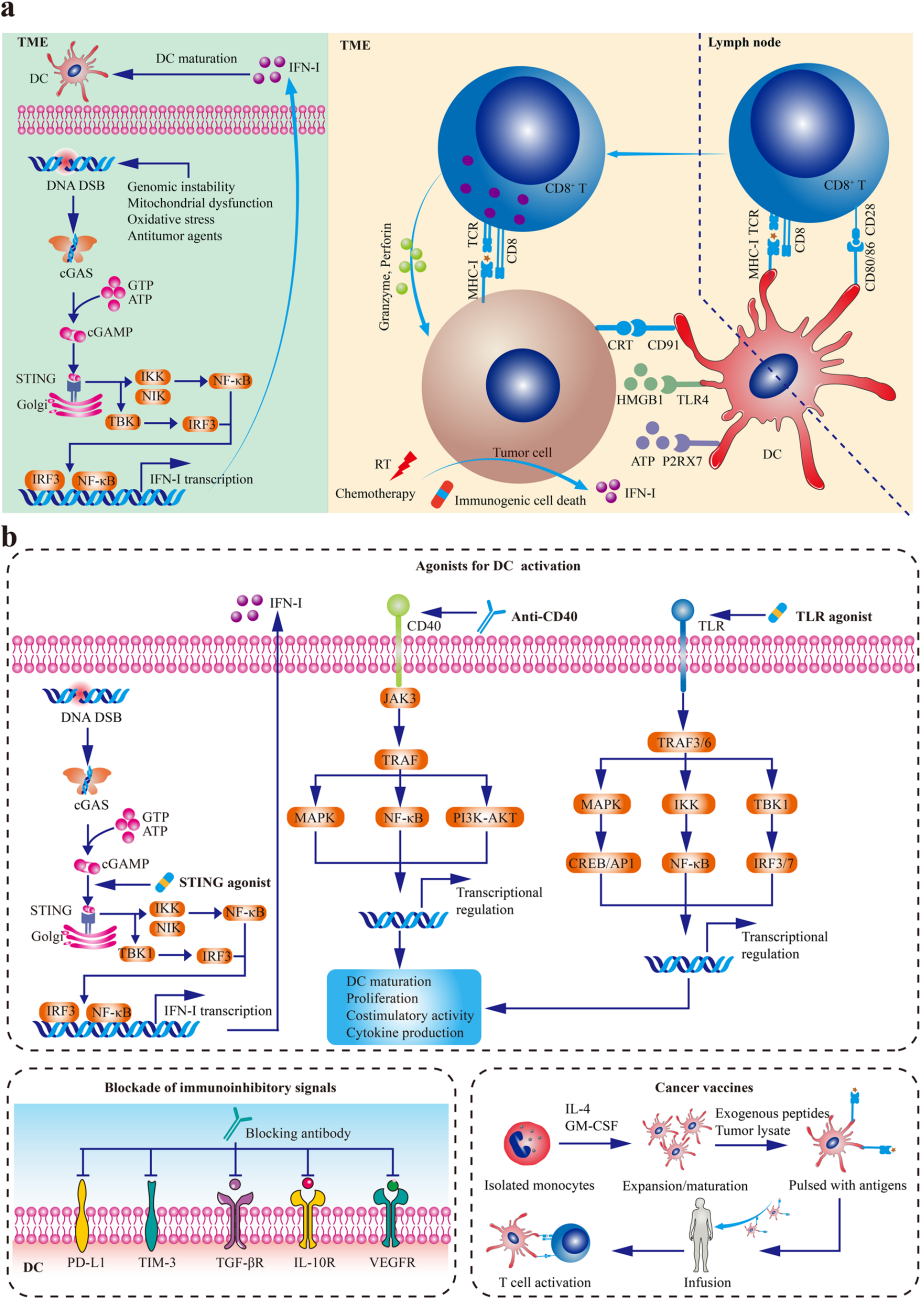
DC-targeted cancer therapies. **a** The maturation of DCs. In the TME, genomic instability, mitochondrial dysfunction, oxidative stress, and conventional antitumor regimens could support DC maturation by inducing DNA damage and activating cytosolic DNA sensing signaling, such as cGAS/STING/IFN-I pathway. Besides, In the presence of damage-associated molecular patterns from stressed or injured cancer cells, these immature DCs are activated by various PRR pathways. Additionally, chemotherapy and radiotherapy could promote DC maturation by inducing the ICD of cancer cells. DAMPs released during ICD stimulate DC maturation and improve DC functions: ATP facilitates DC recruitment and activation, CRT enhances cancer antigen engulfment, and HMGB1 improves antigen presentation of DCs. **b** DC-targeted cancer therapies. DC-targeted strategies mainly consist of agonists for DC differentiation, expansion, and activation, blockade of immunoinhibitory signals, and DC vaccines. Abbreviations: DC, dendritic cell; ICD, immunogenic cell death; ATP, adenosine triphosphate; CRT, calreticulin; HMGB1, high-mobility group box 1. Adapted from Yi et al. 2022 [62].

The presence and accumulation of DCs are the prerequisites of innate immune sensing. The recruitment and expansion of DC in the TME are dependent on several cytokines and chemokines, such as NK cell-derived FLT3L [56], XCL1, CCL5 [57], as well as tumor-derived CCL4 [58]. In the presence of damage-associated molecular patterns (DAMPs) from stressed or injured cancer cells, these immature DCs are activated by various PRR pathways [59]. Additionally, chemotherapy and radiotherapy could promote DC maturation by inducing the immunogenic cell death (ICD) of cancer cells [60]. DAMPs released during ICD stimulate DC maturation and improve DC functions: adenosine triphosphate (ATP) facilitating DC recruitment and activation, calreticulin (CRT) enhancing cancer antigen engulfment, and high-mobility group box 1 (HMGB1) improving antigen presentation of DCs [60]. Moreover, genomic instability, mitochondrial dysfunction, oxidative stress, and conventional antitumor regimens could support DC maturation by inducing DNA damage and activating cytosolic DNA sensing signaling, such as cGAS/STING/IFN-I pathway (Fig. 1a) [61].

Once upon cancer antigen capture, DCs undergo maturation with the licensing stimuli such as IFN-I. In this process, DCs alter their morphology, upregulate costimulatory molecules such as CD40, CD80, and CD86, enhance antigen presentation capability, and secret proinflammatory cytokines [63]. Then, mature DCs migrate to the T cell-rich zone of tdLNs in the CCR7/CCL21-dependent manner [64, 65]. In tdLNs, mature DCs (primarily cDC1s) cross-prime naïve CD8^+^ T cells by DC-T cell immune synapses. Also, cDC1s could prime naïve CD4^+^ T cells by MHC-II, while activated CD4^+^ T cells license cDC1s to trigger cancer-specific CD8^+^ T cell response in turn [66].

Apart from tdLNs, DCs could continue interacting with T cells in the TME to support cancer-specific immunity. Tumor-infiltrating cDC1 promotes T cell infiltration by secreting CXCL9 and CXCL10 (ligands of CXCR3) to guide T cell homing [67]. Beyond de novo T cell priming, tumor-infiltrating CD103^+^ DCs maintain T cell response by restimulating previously activated or memory CD8^+^ T cells [68, 69]. Recent studies demonstrate a positive feedback loop between cDC1s and T cells. After primed and activated by cDC1s, CD8^+^ T cells could secret IFN-γ to promote cDC1s to product IL-12 in a non-canonical NF-κB-dependent manner [70].

### Dysregulated DC functions in the TME

The functions of DCs are disturbed by various immunosuppressive factors in the TME, hampering immune surveillance and supporting tumor progression [71]. Some tumor and stroma-derived cytokines regulate the survival, differentiation, maturation, and antigen presentation of DCs. For example, transforming growth factor-β (TGF-β) is a crucial component in maintaining host immune homeostasis [72]. Deleting *Tgfbr2* in DCs by CD11c-Cre murine models leads to multiorgan inflammation [73]. On the one hand, TGF-β inhibits the antigen-presentation of DCs by downregulating MHC-II expression [74]. Also, the TGF-β-inhibitor of differentiation 1 (ID1) axis induces DC differentiation toward an immunosuppressive myeloid cell phenotype [75]. In murine melanoma and breast cancer models, activated TGF-β signaling increases enzyme indoleamine 2,3-dioxygenase (IDO) in pDCs and CCL22 in myeloid DCs, promoting Treg infiltration as well as immune escape [76]. On the other hand, these tolerogenic DCs contribute to cancer immune evasion by TGF-β secretion. Tumor cells educate DCs to generate TGF-β, which in turn facilitates Treg differentiation [77].

Along with TGF-β, other immunoinhibitory molecules also limit the functions of DCs. IL-10 drives the transformation of immature DCs towards the tolerogenic phenotype [78]. IL-6 undermines DC maturation by STAT3-mediated downregulation of MHC-II and CCR7 [79]. Besides, IL-6 cooperates with prostaglandin E2 (PGE2) to convert cDC2 to the CD14^+^ immunosuppressive phenotype [80]. PGE2 alone could disturb NK cell-stimulated cDC1 recruitment by suppressing NK cell survival and chemokine receptor expression of cDC1 [57]. Moreover, IL-10 inhibits IL-12 production of CD103^+^ cDC1s [81, 82]. Vascular endothelial-derived growth factor (VEGF) is identified as another cytokine hampering the differentiation and antigen presentation of DCs [83–85]. Increased VEGF is associated with decreased circulating and tumor-infiltrating DCs [86]. Some tumor-derived metabolites, such as oxysterols and lactic acid, restrain the CCR7-mediated migration and antigen presentation capability of DCs [87, 88]. Further investigations showed the activation of lactate receptor GPR81 specifically downregulated MHC-II expression [89]. Generally, the functions of DCs are dampened, and antigen presentation machinery is disorganized in the TME [90]. Therefore, reinvigorating DC from abnormal status is feasible to boost antitumor immunity and overcome immunotherapy resistance [91, 92].

### Harnessing DC for cancer treatment

As the core component bridging innate immunity and adaptive immunity, DC is a valuable target for immunotherapy, especially for patients resistant to T cell-based therapies. At present, DC-targeted strategies mainly consist of agonists for DC differentiation, expansion, and activation, blockade of immunoinhibitory signals, and DC vaccines (Fig. 1b) (Table 1) [93].

**Table 1:** Dendritic cell-targeted immunotherapies for cancer patients

Classification	Agent	Target	Therapeutic effects on DCs
Agonists for DC differentiation, expansion, and activation	STING agonist	cGAS-STING pathway	Promoting IFN-I production, DC maturation, antigen presentation, and cross-priming of T cells
	TLR2/4 agonists	TLR2/4	Mainly promoting cDC2 activation
	TLR3 agonists	TLR3	Mainly promoting cDC1 activation
	TLR7/8 agonists	TLR7/8	Promoting pDC and cDC activation
	TLR9 agonists	TLR9	Promoting pDC and cDC activation
	FLT3L	Flt3-FLT3L	Expanding cDC
	GM-CSF	GM-CSF-GMR	Promoting cDC moblization and activation
	RIG-I agonists	RIG-I-MAVS pathway	Enhancing DC phagocytic potential
	Agonistic CD40 antibodies	CD40L-CD40	Enhancing cross-priming of T cells and educating macrophage to degenerate fibrosis
Blockade of immunoinhibitory signals	VEGF inhibitors	VEGF-VEGFR pathway	Increasing functional DCs in the TME
	Anti-IL-10 receptor antibodies	IL-10 receptor pathway	Increasing IL-12 production
	Anti-TGF-β antibodies	TGF-β signaling pathway	Increasing functional DCs in the TME
	Anti-PD-L1 antibodies	PD-L1-PD1 and PD-L1-CD28 interactions	Reactivating dysfunctional T cells inside tumors and allowing CD80/CD28 interaction to provide costimulatory signaling for T cell activation
	Anti-TIM-3 antibodies	TIM-3	Promoting the activation of the cGAS-STING pathway and CXCL9 expression in cDC1
Cancer vaccines	Tumor-associated antigens or neoantigens	Tumor antigens	Improving cancer-specific adaptive immune response
	DC vaccines	Autologous cDC precursors or monocyte-derived DCs loaded with cancer antigens	Improving cancer-specific adaptive immune response

*DC* dendritic cell, *STING* stimulator of interferon genes, *IFN-I* type I interferon, *TLR* toll-like receptor, *Flt3L* Fms-like tyrokine kinase 3 ligand, *GM-CSF* granulocyte–macrophage colony-stimulating factor, *GMR* GM-CSF receptor, *RIG-I* retinoic acid inducible gene I, *VEGF* vascular endothelial-derived growth factor, *TGF-β* transforming growth factor-β, *PD-1* programmed death-protein 1, *PD-L1* programmed death ligand 1, *TIM-3* T cell immunoglobulin and mucin-domain containing-3

#### Agonists for DC, differentiation, expansion, and activation

The cGAS/STING signaling is a well-known innate immune sensing mechanism responding to infection, senescence, DNA damage, and dysregulated cell cycle [94]. cGAS recognizes cytoplasmic double-stranded DNA and then catalyzes the formation of secondary messenger cyclic GMP-AMP (cGAMP). Stimulated by cGAMP, STING undergoes conformation changes and then translocates from endoplasmic reticulum to Golgi body, triggering downstream TBK1/IRF3/IFN-I or TBK1/NF-κB cascades [61, 95]. STING-dependent TBK1/IRF3/IFN-I axis licenses DCs to cross-present cancer antigens to CD8^+^ T cells with MHC-I molecules. At the same time, STING-dependent NF-κB activation enables DCs to generate proinflammatory cytokines. Notably, in some tumor-associated myeloid cells, STING-dependent NF-κB signaling could also be initialized by inhibitor of kB kinase ε (IKK-ε) in a TBK1-independent manner [96]. Based on the immunostimulatory effects of STING-dependent IFN-I production, pharmacological activation of STING by intratumorally injecting cGAMP retards tumor growth in multiple murine colon carcinoma and melanoma models [97–101]. However, the applications of cGAMP and synthetic cyclic dinucleotides (CDNs) are limited by poor bioavailability and intratumoral delivery [102]. Relatively, non-CDN small-molecule STING agonists overcome these shortcomings that could be systemically delivered. Despite the failure of DMXAA [103], some novel STING agonists, such as di-ABZI, MSA-2, and manganese, exhibit potent antitumor activity in murine tumor models, which are undergoing clinical evaluations (Table 2) [104–108]. These STING agonists effectively upregulate costimulatory molecules (e.g., CD40, CD80, CD83, and CD86) and MHC on DCs. Besides, STING agonists improve the antigen presentation of DCs, especially the tumor-specific antigen cross-presentation to CD8^+^ T cells [95]. As a result, STING agonist administration enhances the expression of IFN-β and other proinflammatory cytokines (e.g., IL-6 and TNF-α) or chemokines (e.g., CCL2/3/4/5 and CXCL9/10), the maturation and functions of DCs, and the expansion of tumor-infiltrating CD8^+^ T cells [106]. Besides, some STING agonists, such as manganese, could strengthen NK cell activation and NK cell-mediated cytotoxicity in the TME [107]. STING agonists are a promising strategy for cancer immunotherapy, mobilizing the innate defensive sensor for immunological surveillance and promoting cancer-specific T cell priming.

**Table 2:** STING agonists for cancer immunotherapy

Agents	Delivery	Molecular Type	Combination therapy	Clinical trials	Cancer types	Phase
ADU-S100	IT	CDN analog	Pembrolizumab	NCT03937141	Advanced head and neck cancer	2
			Ipilimumab	NCT02675439	Advanced solid tumors or lymphomas	1
			PDR001 (Anti-PD-1)	NCT03172936	Advanced solid tumors or lymphomas	1
MK-1454	IT	CDN analog	Pembrolizumab	NCT04220866	Advanced head and neck cancer	2
			Pembrolizumab	NCT03010176	Advanced solid tumors or lymphomas	1
MK-2118	IT or SC	Non-CDN	Pembrolizumab	NCT03249792	Advanced solid tumors or lymphomas	1
SB11285	IV	CDN analog	Atezolizumab	NCT04096638	Advanced solid tumors	1
GSK3745417	IV	Non-CDN	NA (Monotherapy)	NCT05424380	Refractory myeloid malignancies	1
			Dostarlimab	NCT03843359	Advanced solid tumors	1
BMS-986301	IM or IV or IT	CDN analog	Nivolumab or Ipilimumab	NCT03956680	Advanced solid tumors	1
BI 1387446	IT	CDN analog	Ezabenlimab	NCT04147234	Advanced solid tumors	1
E7766	IT	Non-CDN	NA (Monotherapy)	NCT04144140	Advanced solid tumors or lymphomas	1
			NA (Monotherapy)	NCT04109092	Bladder cancer	1
TAK-676	IV	CDN analog	Pembrolizumab	NCT04879849	Advanced solid tumors	1
			Pembrolizumab	NCT04420884	Advanced solid tumors	1
SNX281	IV	Non-CDN	Pembrolizumab	NCT04609579	Advanced solid tumors or lymphomas	1
SYNB1891	IT	Engineered bacteria vectors	Atezolizumab	NCT04167137	Advanced solid tumors or lymphomas	1
Manganese	Inhalation	Non-CDN	Radiotherapy	NCT04873440	Advanced solid tumors or lymphomas	1/2

*CDN* cyclic dinucleotide, *IT* intratumoral, *IM* intramuscular, *IV* intravenous, *SC* subcutaneous

Besides cGAS/STING, Toll-like receptors (TLRs) are also damage- or pathogen-sensing pathways contributing to DC activation [109]. Up to now, more than ten functional TLRs (TLR1-10) have been identified in humans [110]. Human DC subsets have different TLR expression patterns: TLR3/8 in cDC1 and TLR7/9 in pDC [93, 111]. TLR3 agonist such as Poly(I:C) enhances cDC1 maturation and cytokine production such as IL-12 and IFN-I [112]. Additionally, TLR8 agonist, such as Motolimod, promotes cDC1 maturation, with encouraging antitumor activity and tolerable toxicity profiles in squamous cell head and neck cancer [113–115]. Moreover, TLR7 and TLR9 are widely explored due to their capability to induce IFN-I generation in pDCs. The immunostimulatory effects and antitumor activity of TLR7 agonists such as Imiquimod have been confirmed in various types of cancer [116–119]. TLR9 agonists also promote cytokine production and pDC maturation [120, 121]. Also, other novel agents such as granulocyte macrophage-colony stimulating factor (GM-CSF), Flt3L agonist, and RIG-I agonist improve DC-mediated T cell response by expanding DC population, promoting DC activation or phagocytic potential [122–125].

In contrast with the co-inhibitory signaling pathway, costimulatory pathways such as CD40/CD40L enhance the cross-priming capability of antigen-presenting cells [13]. CD40 on DCs is activated by CD40L on CD4^+^ T cells, leading to the upregulation of MHC, costimulatory molecules, and various TNF superfamily ligands (CD137L, GITRL, and OX40L). Furthermore, CD40-activated DCs generate more IL-12 to support CD8^+^ T cell activation and skew the following adaptive immunity toward Th1 polarization [126]. Overwhelming evidence demonstrates that agonistic CD40 antibodies expand cancer antigen-specific CD8^+^ T cells and provide robust immune protection by cross-presenting DCs [127]. In some murine tumor models, the antitumor activity of agonistic CD40 antibodies is T cell-dependent [128–130]. However, some current studies showed that CD40 activation-mediated tumor regression was independent of T cells. On the contrary, agonistic CD40 antibodies activate macrophages (also highly expressing CD40), causing stroma depletion and tumor regressions [131]. This effect is attributed to systemically released IFNγ and CCL2, which redirect Ly6C^+^CCR2^+^ monocytes and macrophages to infiltrate into the TME and degenerate fibrosis [132]. To date, multiple CD40-targeted monoclonal antibodies have been developed and tested in clinical trials [133]. Generally, agonistic CD40 antibodies have a minimal response rate in cancer patients, except for selicrelumab [134]. In the phase 1 study of selicrelumab, 27% of melanoma patients achieved partial responses [134, 135]. For most types of cancers with low immunogenicity, it is hard to effectively destroy tumors by agonistic CD40 antibody monotherapy. Combination therapies with chemotherapy, radiotherapy, or other immunotherapies might be worth exploring in the future [126].

#### Blockade of immunoinhibitory signals

As mentioned above, various immunosuppressive factors like TGF-β, IL-10, IDO, PGE2, and VEGF hamper the functions of DCs, hindering immune surveillance and promoting tumor advancement [93, 136]. Therefore, neutralizing these immunoinhibitory factors enhances the recruitment, survival, activation, and antigen presentation capability of DCs [137]. Anti-VEGF antibodies improve the functions of DCs of spleen and lymph node, synergizing with peptide-pulsed DCs to prolong the survival of tumor-bearing mice [138]. In a phase 1 study of VEGF-Trap, VEGF inhibition significantly increased the ratio of mature DCs, without alterations in populations of total DCs [139]. Besides, in the MMTV-PyMT tumor model, blocking IL-10 signaling by anti-IL-10 receptor antibody enhanced treatment response to carboplatin and paclitaxel. This improved efficacy is attributed to the strengthened IL-12 production of DC and CD8^+^ T cell response [82]. Also, neutralizing TGF-β by conventional or bispecific antibodies increases the number of functional DCs in the TME [140–142]. Furthermore, IDO, functioning as an intracellular enzyme within the cytosol, transforms tryptophan into kynurenine. This conversion disrupts the activities of cytotoxic T cells, elevates the presence of Tregs and TAMs, and impedes the maturation of DCs [143–145]. Consequently, IDO contributes to rendering the TME more immunosuppressive, facilitating cancer escape from immune surveillance. Pharmacologic inhibition of IDO or deletion of *Ido1* gene induces differentiation of inflammatory Ly6c^+^CD103^+^ DCs in mice, promoting anti-tumor T-cell response and inhibiting tumor growth [146]. The application of anti-IDO siRNA therapy enhances cytokine production and the antigen presentation capabilities of DCs [147]. Tumor vaccines that incorporate IDO inhibitors effectively enhance the uptake of tumor antigens and the maturation of DCs, ultimately inducing a robust tumor-specific T-cell response [148]. Currently, numerous clinical trials are underway to assess the effectiveness of immunotherapies involving IDO inhibitors [144].

Recent data demonstrate that PD-L1 on DCs dampens T cell activation and antitumor immune response. PD-L1 blockade enhances de novo T cell priming in tdLNs and reactivates dysfunctional T cells in the TME [149]. The antitumor activity of anti-PD-L1 therapy is more dependent on the renaissance of dysfunctional T cells inside tumors rather than newly activated T cell response in tdLNs [149]. Moreover, DCs could simultaneously overexpress PD-1, PD-L1, and CD80 [150, 151]. When DCs express a large amount of CD80, the cis-CD80/PD-L1 interactions on DCs prevent PD-L1 binding to PD-1 on T cells, contributing to the optimal T cell response [152]. However, for patients with cancers, the expression level of PD-L1 is significantly higher than CD80 on tumor-associated and peripheral DCs [153]. In this situation, anti-PD-L1 antibodies dissociate cis-CD80/PD-L1 binding, allowing CD80/CD28 interaction to provide costimulatory signaling for T cell activation [153]. Apart from PD-L1, T-cell immunoglobulin and mucin domain 3 (TIM-3) expressed on tumor-infiltrating DCs suppresses HMGB1-mediated activation of the innate sensing system [154]. Further explorations reveal that TIM-3 limits HMGB1-dependent DNA uptake, while TIM-3 blockade promotes the activation of the cGAS-STING pathway and CXCL9 expression of cDC1 [155]. Extensive preclinical evidence has demonstrated the advantages of anti-TIM-3 antibodies, especially in combination with anti-PD-1/PD-L1 therapies [156]. The therapeutic potential of TIM-3 blockade is currently being evaluated in multiple types of cancers.

#### Cancer vaccines and other strategies

The administration of cancer antigens, which could be captured and presented by endogenous DCs, is a promising immunotherapy approach [157]. These cancer antigen vaccines contain synthetic peptides, recombinant cancer antigen-expressing viruses, or tumor lysates [55, 158]. Fuelled by next-generation sequencing and prediction algorithms in silico, the identification of neoantigens increases the specificity of cancer antigen vaccines [159–161]. Considering that antigen presentation by DCs is the cornerstone for cancer antigen vaccines, antigens and adjuvants are usually encapsulated in degradable biomaterial or nanoparticles [162, 163]. To date, YS-ON-001 (rabies virus-based vaccine) has been approved for pancreatic cancer and hepatocellular carcinoma in the US [164]. Currently, advances have been made in targeted delivery to specific DC subsets [165]. DEC205, langerin, and CLEC9A are commonly used to target cDC1s. In vitro experiments confirm that the fusion protein of anti-DEC205 single-chain fragment variable and peptides of cancer antigen MAGE-A3 is presented more efficiently than direct peptide pulse [166]. Fusion antibody of anti-DEC205 and cancer antigen NY-ESO-1 effectively mobilizes CD8^+^ T cell response [167], showing encouraging antitumor activity in phase 1 studies [168]. Besides, more DC-targeted cancer antigen vaccines, such as CD209/DC-SIGN-fusion protein, are still under evaluation [169–171].

In addition to cancer antigen vaccines, the application of DC vaccines is extensively explored as well (Table 3) [157]. Such vaccines consist of manipulated autologous DCs isolated from cancer patients and expanded in vitro [172]. cDC precursors or monocyte-derived DCs are loaded with cancer antigens, activated with cytokine cocktails, and then reinfused into patients [173]. In various types of cancers, including non-small cell lung cancer (NSCLC), ovarian cancer, prostate cancer, melanoma, renal cell carcinoma, and glioblastoma, DC vaccines exhibit potent antitumor activity with a manageable safety profile [174–184]. In the latest phase 3 study of tumor lysate-loaded DC vaccine (DCVax-L), the combination of DCVax-L and standard of care (temozolomide) significantly extended the survival of patients with recurrent (HR = 0.58; *P* < 0.001) or newly diagnosed (HR = 0.80; *P* = 0.002) glioblastoma, compared to patients receiving temozolomide treatment alone [182]. At present, the DC vaccine sipuleucel-T (consisting of autologous DCs pulsed with the recombinant fusion protein containing GM-CSF and prostatic acid phosphatase) has been approved for prostate cancer [185]. In the phase 3 study NCT00065442, sipuleucel-T prolonged the survival of patients with castration-resistant prostate cancer (HR = 0.77; *P* = 0.02) [186]. However, immunosuppressive TME is a great obstacle to DC vaccination. Thus, the combination of DC vaccination with other therapies, such as immune checkpoint inhibitors, appears ideal for fostering de novo cancer-specific T-cell response [187].

**Table 3:** Representative clinical studies of dendritic cell vaccines for cancer immunotherapy

Clinical trials	Cancer types	DC vaccines	Phase	Status
NCT00006434	Non-Hodgkin’s Lymphoma	Tumor lysate-pulsed DCs	3	Completed
NCT03905902	Ovarian cancer, fallopian tube cancer, peritoneal carcinoma	Autologous DCs (DCVAC/OvCa)	3	Withdrawn
NCT00779402	Prostate cancer	PAP-loaded DC vaccine (Sipuleucel-T)	3	Completed
NCT05100641	Glioblastoma	Therapeutic autologous DC vaccine (AV-GBM-1)	3	Not yet recruiting
NCT02503150	Colorectal cancer	Antigen-pulsed DCs	3	Unknown
NCT04277221	Glioblastoma	Autologous DC/tumor antigen	3	Unknown
NCT00005947	Prostate cancer	PAP-loaded DC vaccine (Sipuleucel-T)	3	Completed
NCT01759810	Glioblastoma	Proteome-based DC vaccine	3	Unknown
NCT01782287	Lung cancer brain metastases	Proteome-based DC vaccine	3	Unknown
NCT01983748	Uveal melanoma	Autologous DCs loaded with autologous tumor RNA	3	Active, not recruiting
NCT04348747	Brain metastasis from TNBC or HER2^+^ breast cancer	Anti-HER2/HER3 DC vaccine	2	Recruiting
NCT05127824	Kidney cancer	Autologous alpha-DC1/TBVA vaccine	2	Not yet recruiting
NCT04912765	Hepatocellular carcinoma or liver metastases From colorectal cancer	Neoantigen-loaded DC vaccine	2	Recruiting
NCT01876212	Melanoma	Type I-polarized autologous DC vaccine	2	Completed
NCT02285413	Melanoma	Mature DC loaded with mRNA encoding tumor-associated antigens gp100 and tyrosinase	2	Completed
NCT00266110	Breast cancer	Therapeutic autologous DCs	2	Completed
NCT02362464	Prostate cancer	Multi-epitope TARP peptide autologous DC vaccine	2	Completed
NCT01413295	Colorectal cancer	Autologous DCs loaded with autologous tumor antigens	2	Completed
NCT04487756	Lung cancer	Autologous DC vaccine	1/2	Recruiting
NCT02061332	Breast cancer	HER-2 pulsed DC vaccine	1/2	Completed
NCT00087984	Kidney cancer	RNA-loaded DC vaccine	1/2	Completed

*TNBC* triple-negative breast cancer, *DC* dendritic cell, *TARP* T-cell receptor gamma chain alternate reading frame protein

Other DC-targeted strategies, such as agents improving DC migration by the CCR7-CCL19/CCL21 axis, have been adopted for cancer immunotherapy. When DCs encounter foreign stimuli, they undergo a mature process, with the upregulation of costimulatory molecules, MHC, and CCR7. The increased CCR7 expression on DCs drives their migration toward lymph nodes under the guide of the CCL19/CCL21 concentration gradient. Then, the CCR7-CCL19/CCL21 signaling directs DCs to distribute in the T-cell zone, where they prime and activate naïve T cells by antigen presentation [188]. Theoretically, CCL19 or CCL21 therapy could potentiate antitumor immunity by improving the trafficking of cytotoxic T cells and DCs. In multiple murine tumor models, intratumoral injection of CCL19 or CCL21 increases the numbers of tumor-infiltrating DCs and T cells, retards tumor growth, and prolongs the survival of tumor-bearing mice [189–193]. Besides, inducing tumor cells to overexpress CCL19 or CCL21 by transfection also enhances the functions of DCs and tumor control [194–197]. Also, autologous DCs engineered to overexpress CCR7 exhibit stronger migration capability and antitumor properties in murine tumors [198]. Besides immune response, CCR7 signaling also contributes to tumor progression, especially metastasis to the lymph nodes [199]. As a result, approaches inhibiting lymph node metastasis through CCR7 antagonism might unintentionally hinder the immune response to cancer. Conversely, strategies enhancing CCR7 expression or introducing CCL19/CCL21 into the TME could inadvertently promote metastasis. Therefore, several unresolved questions remain, necessitating answers before maximizing the therapeutic potential of the CCL19/CCL21-CCR7 axis. The initial pivotal question stems from the paradox between CCR7’s roles in enhancing the immune response to tumors and facilitating lymph node migration and metastasis.

Besides, IL-12 is a proinflammatory cytokine that activates both the innate and adaptive arms of the host immune system. In preclinical investigations, recombinant IL-12 has demonstrated strong antitumor effects [200]. It has been observed that the success of anti-PD-1 therapy relies on the presence of IL-12-producing DCs [70]. To address the challenges associated with the toxicity of systemic IL-12 administration, various localized delivery methods for IL-12 have been developed. These approaches include immunocytokine fusion, cell-based delivery, nucleic acid-based delivery, and virus-based delivery [201–204]. Clinical studies have confirmed the safety of intratumoral injections involving an adenoviral vector encoding IL-12 or DCs transfected with an adenovirus encoding IL-12 [205, 206]. Additionally, virotherapy through the intratumoral injection of a Semliki Forest virus encoding IL-12 (SFV-IL-12) has been shown to induce an inflammatory response and synergize effectively with anti-PD-1 therapy in tumor models [207]. Furthermore, SFV-IL-12 has been found to enhance the therapeutic effects of a 4-1BB agonist antibody [208]. In multiple preclinical investigations, the adoptive transfer of tumor-specific CD8^+^ T cells transiently expressing IL-12 has also demonstrated significant antitumor activity [209, 210]. Together, the outcomes of localized IL-12 immunotherapies, particularly in preclinical studies, have shown significant potential, meriting further investigation in clinical studies.

## Macrophage-targeted cancer immunotherapy

Macrophages are a heterogeneous population of cells with high plasticity, showing diverse phenotypes under different stimuli [211]. Historically, macrophages are classified into two phenotypes, commonly referred to as M1 (classically activation, stimulated by IFN-γ and TLR ligands) and M2 (alternatively activation, stimulated by IL-4 and IL-13) [212]. M1 phenotype contributes to macrophage-mediated inflammatory tissue injury and tumor cell clearance, while M2 phenotype participates in damage repair and remodeling, as well as defense against parasites [213]. In the process of inflammation, the activation and polarization of macrophages are dynamically changed: M1 cells in triggering and propagating immune response, M2 or M2-like populations in inflammation resolution, or smouldering chronic inflammation [214–216]. However, with the development of omics technology, more and more novel macrophage subsets have been identified, and mixed expression of M1 and M2 biomarkers is also observed in tumor-infiltrating macrophages [217–220]. It is realized that the M1-M2 classification system is too simplistic to present complex phenotypes of macrophages.

### Tumor-associated macrophage (TAM)

Tumor-infiltrating macrophage (termed TAM) is an important player in antitumor immune response and cancer progression [221]. Although some studies have opposite results [222], high infiltration of TAM is generally considered a risk factor in most preclinical and clinical studies [223]. Notably, signals regulating the polarization and education of TAMs change in different tumors and even in different stages or spatial locations of the same tumor, leading to various phenotypes of TAMs [224–226]. Therefore, TAM subsets should be preciously redefined to elaborate on the distinct roles of TAMs under specific circumstances.

**Fig. 2:**
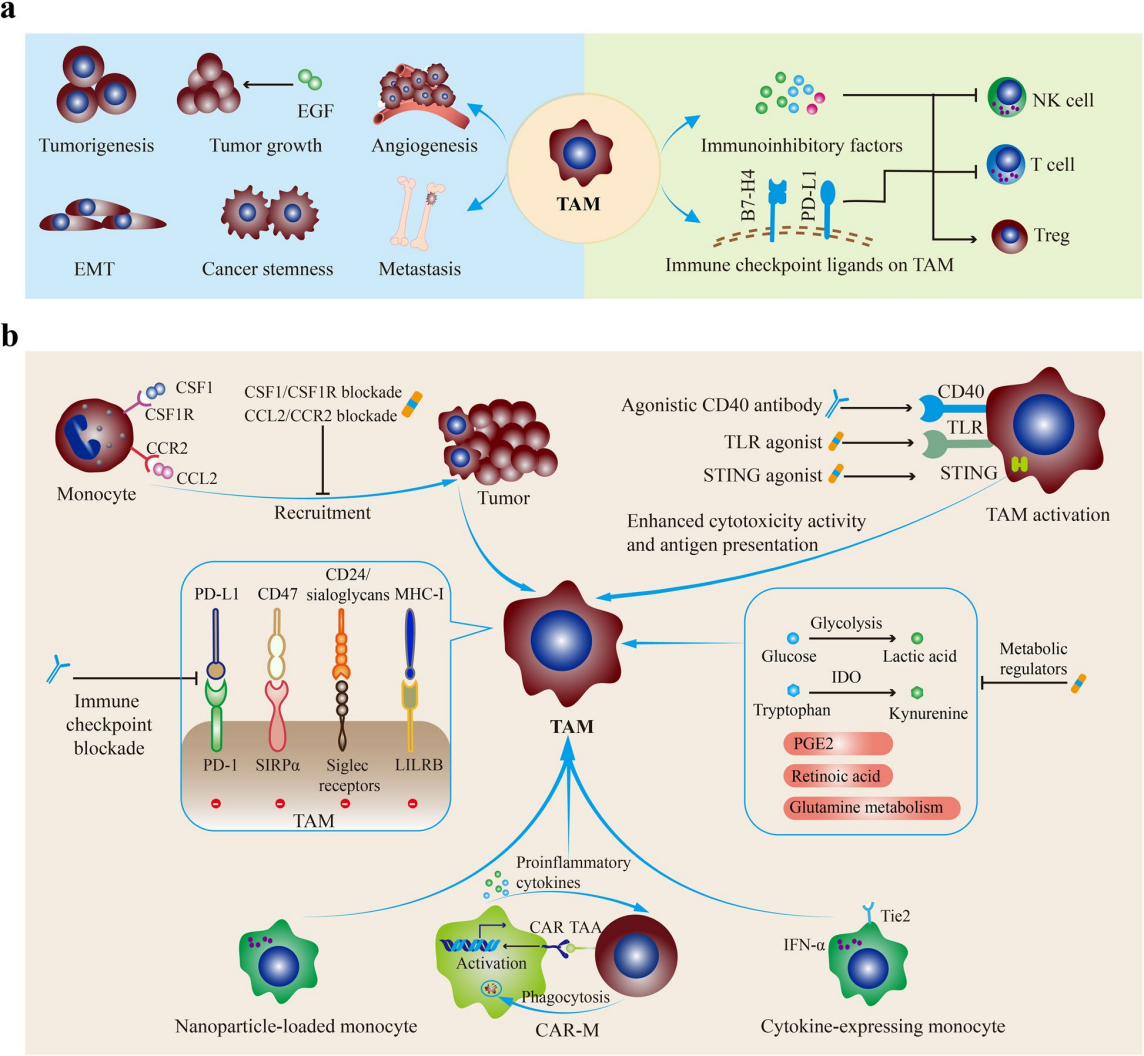
The protumor activities of TAMs and TAM-targeted cancer therapies. **a** The protumor properties of TAMs. TAMs are commonly set in the protumor M2-like phenotype and have substantial influences on tumor initiation and progression. On the one hand, TAM-derived soluble molecules directly suppress the functions of tumor-infiltrating T cells and NK cells. Besides, autocrine IL-10 and TNF-α stimulate PD-L1 upregulation on TAMs. Also, TAMs directly suppress the antitumor immune response by recruiting Tregs and supporting their differentiation. On the other hand, TAMs also promote tumor progression in immune-independent ways, including tumor initiation and growth, angiogenesis, stemness, EMT, and distant metastasis. **b** TAM-targeted therapies. TAMs could be harnessed by targeting their recruitment, activation, immune checkpoint pathways, and metabolism. Besides, macrophage-based cell therapies, such as nanoparticle-loaded monocytes, CAR-M, and genetically engineered hematopoietic progenitors, also show potent antitumor activities. Abbreviations: TAM, tumor-associated macrophage; EMT, epithelial-mesenchymal transition; CSF1, colony-stimulating factor 1; CSF1R, CSF1 receptor; TLR, Toll-like receptor; STING, Stimulator of interferon genes; LILRB, Leukocyte immunoglobulin-like receptor B; SIRPα, Signal regulatory protein-α; IDO, Indoleamine 2,3-dioxygenase; CAR, Chimeric antigen receptor

Macrophages are recruited and educated by multiple factors in the TME, including colony-stimulating factor-1 (CSF1), GM-CSF, TGF-β, IL-1, IL-4, CCL2, CCL5, immune complexes, complement, histamine, tumor-derived non-coding RNAs [213, 227–230]. Besides, increased TNF-α and IL-1β in the TME could induce IL-8 expression, which recruits immunosuppressive myeloid leukocytes including macrophages and predicts poor outcomes in patients treated with immune checkpoint inhibitors [231, 232]. As a result, TAMs are commonly set in the protumor M2-like phenotype [233]. It has been validated that TAMs have substantial influences on tumor initiation and progression, especially by enhancing immune escape [234–239]. TAM-derived soluble molecules such as IL-10, IL-23, TGF-β, IDO, PGE2, and arginase 1 (ARG1) directly suppress the functions of tumor-infiltrating T and NK cells (Fig. 2a) [240–245]. Besides, autocrine IL-10 and TNF-α stimulate PD-L1 upregulation on TAMs [246]. These increased immune checkpoint ligands, such as B7-H4 and PD-L1/2, induce T cell exhaustion [247, 248]. Furthermore, TAMs inhibit the functions of T cells and NK cells by HLA-G/ILT2 and HLA-E/CD94 pathways [249]. Also, TAMs directly suppress the antitumor immune response by recruiting Tregs and supporting their differentiation [250]. Chemokines produced by TAMs, including CCL5, CCL20, and CCL22, recruit Treg into TME, while TGF-β and IL-10 induce Treg differentiation [249, 250].

In parallel with the immunosuppressive effects, TAMs also promote tumor progression in immune-independent ways, including angiogenesis, stemness, treatment resistance, and distant metastasis [251]. In gastric and colon cancers, chronic inflammation and oncogenic signals enhance the activities of multiple inflammation-associated transcription factors such as NF-κB, STAT3, and HIF-1α, recruiting macrophages into the TME [211]. Subsequently, these recruited macrophages generate a panel of molecules (e.g., EGF, proinflammatory cytokines, and ROS) to reshape the microenvironment and facilitate tumor initiation [252–256]. Also, TAMs induce epithelial-mesenchymal transition (EMT) of cancer cells by secreting CCL2, CCL5, CCL18, COX-2, MMP9, EGF, TGF-β, and IL-6 [254, 257–263]. These paracrine cytokines from TAMs endow cancers with greater invasive and metastatic capacities [264]. Furthermore, TAM-derived soluble molecules and TAM-tumor interactions maintain the stemness of cancer cells [265–268]. Moreover, TAMs support tumor growth by producing proangiogenic factors, including VEGFA, EGF, and TGF-β1 [269–271]. Given the pivotal roles of TAMs in cancer development, intensive attempts have been made to delete TAMs or reprogram TAM behaviors.

### TAM-targeted therapies

Numerous studies have confirmed the protumor roles of TAM in the majority of human tumors. As a result, targeting TAMs has emerged as a promising therapeutic strategy for cancer patients (Table 4). In the ensuing paragraphs, we summarize several TAM-based therapeutic strategies, including targeting TAM recruitment, activation, and metabolism (Fig. 2b). Besides, myeloid checkpoint inhibitors and macrophage cell therapies are promising, especially with present immune checkpoint blockade.

**Table 4:** Macrophage-targeted immunotherapies for cancer patients

Classification	Target	Agent	Representative clinical trials	Cancer type	Phase
Inhibiting TAM recruitment and expansion	CCL2	Carlumab	NCT00992186	Prostate cancer	2
		Carlumab	NCT01204996	Solid tumors	1
		Carlumab	NCT00537368	Solid tumors	1
		Trabectedin	NCT03085225	Soft-tissue sarcomas and ovarian carcinomas	1
	CCR2	PF-04136309	NCT01413022	Pancreatic neoplasms	1
		MLN1202	NCT01015560	Bone metastases	2
	CSF-1	MCS110	NCT02435680	TNBC	2
			NCT00757757	Prostate cancer, Bone Metastases	1/2
	CSF-1R	IMC-CS4	NCT01346358	Solid tumors	1
		AMG 820	NCT01444404	Solid tumors	1
		Emactuzumab	NCT02323191	Solid tumors	1
		ARRY-382	NCT02880371	Solid tumors	1/2
		Pexidartinib	NCT02777710	Pancreatic and colorectal cancers	1
		SNDX-6352	NCT03238027	Solid tumors	1
		BLZ945	NCT02829723	Solid tumors	1/2
		Cabiralizumab	NCT03158272	Malignancies	1
		PLX7486	NCT01804530	Solid tumors	1
Regulating TAM activation	CD40	CP-870,893	NCT02225002	Advanced solid tumors	1
			NCT01103635	Melanoma	1
			NCT00607048	Neoplasms	1
			NCT01456585	Adenocarcinoma pancreas	1
		RO7009789	NCT02760797	Neoplasms	1
			NCT02588443	Pancreatic cancer	1
			NCT02665416	Solid tumors	1
	TLR7/8	NKTR-262	NCT03435640	Solid tumors	1
	STING	ADU-S100	NCT02675439	Advanced solid tumors and lymphomas	1
Targeting immune checkpoints	CD47	Hu5F9-G4	NCT03922477	Acute myeloid leukemia	1
		TTI-621	NCT02663518	Hematologic malignancies and solid tumors	1
		CC-90002	NCT02641002	Acute myeloid leukemia	1
	LILRB2	JTX 8064	NCT04669899	Solid tumors	1/2
		IO-108	NCT05054348	Solid tumors	1
TAM metabolism regulators	Glucose metabolism	2-Deoxyglucose	NCT0063308	Advanced cancer and hormone refractory prostate cancer	1/2
Macrophage cell therapy	Her-2	CT-0508	NCT04660929	Her-2^+^ tumors	1
	IFN-α2	TEMFERON	NCT03866109	Glioblastoma	1/2

*TNBC* triple-negative breast cancer, *CSF1R* colony-stimulating factor 1 receptor, *TLR* toll-like receptor, *STING* stimulator of interferon genes, *LILRB2* leukocyte immunoglobulin like receptor B2

#### Inhibiting TAM recruitment and expansion

As mentioned above, TAM recruitment is driven by chemokines and CSF1. Although therapeutic antibodies or inhibitors targeting attractants such as CCL2-CCR2 (e.g., Lenalidomide and Trabectedin) have exhibited antitumor activities in preclinical studies, there are rare clinical trials with positive data [272]. Relatively, clinical trials of CSF1-CSF1R inhibitors (e.g., Cabiralizumab and Pexidartinib) are experiencing improved efficiency and progress. CSF1-CSF1R blockade deletes the TAM population, retards tumor growth, and increases treatment sensitivity [273–275]. Besides, the CSF1R inhibitor BLZ945 could reprogram TAM from a tumor-promoting toward a tumor-suppressing phenotype, enhancing antigen presentation and T or NK cell activation [273]. Moreover, in the phase 1 study of diffuse-type tenosynovial giant-cell tumor (NCT01494688), anti-CSF1R antibody emactuzumab decreased tumor-infiltrating CD68/CD163^+^ macrophages and achieved pronounced activity (response rate: 71%) [276]. At present, more clinical studies of CSF1R inhibitors combined with other therapies are still ongoing. Some novel TAM depletion strategies, such as CAR-T cells recognizing folate receptor-β, eliminate M2-like TAM subsets and promote tumor-specific T-cell response [277]. Furthermore, Lurbinectedin, which is a synthetic alkaloid, remodels the TME by prompting apoptosis in TAMs and diminishing the expression of CCL2. Lurbinectedin has received approval for the treatment of small-cell lung cancer [278]. Also, there have been recent advancements in the use of M2 macrophage-targeting peptides (M2peps) to specifically target and deliver pro-apoptotic agents to M2-like TAMs in preclinical tumor models [279]. These therapeutic agents associated with M2peps demonstrate preferential toxicity towards M2-like TAMs and exhibit potent anti-tumor effects, holding promise for potential clinical applications in TAM-focused immunomodulation [280].

#### Regulating TAM activation

Classical activation endows macrophages with antitumor properties. Agents enhancing classical activation pathways, including CD40, STING, and TLR, reset TAMs in the antitumor M1-like phenotype. As described above, CD40L-CD40 is the core pathway to activate antigen-presentation cells [281]. Preclinical studies demonstrate that agonistic CD40 antibodies effectively arm macrophages with cytostatic activity against tumor cells, stimulating antitumor response and slowing tumor growth [282, 283]. Furthermore, agonistic CD40 antibodies improve the antigen presentation capability of TAMs by upregulating costimulatory molecules and MHC expression [213]. Besides CD40, agents targeting TLR exert immunostimulatory effects by enhancing the cytotoxic activity and chemokine production of TAMs [284, 285]. The TLR4 agonist monophosphoryl lipid A combined with IFN-γ drives the transformation from CD206^+^ TAMs to iNOS^+^ macrophages, activating T cells by inducing macrophages to secret IL-12 and TNF-α [285]. Additionally, STING agonists promote IFN-I secretion and macrophage polarization toward the M1-like phenotype. In murine tumor models, STING agonists increase the ratio of M1/M2 ratio and synergize with anti-PD-1/PD-L1 therapies [105, 106, 286].

#### Targeting immune checkpoints

The phagocytosis and cross-presentation capabilities of TAMs are constrained by immune checkpoints such as signal regulatory protein-α (SIRPα), SLAM family receptors (SFRs), sialic acid-binding immunoglobulin-like lectin (Siglec), and leukocyte immunoglobulin-like receptor B (LILRB) families [287–289]. CD47 is the ligand of SIRPα, also known as the “not eat me” signal. In the TME, overexpressed CD47 on cancer cells bind to SIRPα on myeloid cells, especially macrophages, monocytes, granulocytes, and CD4^+^ DCs, limiting phagocytosis and intracellular degradation [290]. Agents blocking the CD47-SIRPα axis improve macrophage phagocytosis, enhance programmed cell death of cancer cells, and promote macrophage-mediated ADCP or ADCC effects [291–296]. Besides, anti-CD47 antibody-mediated phagocytosis facilitates antigen presentation and cross-priming of CD8^+^ T cells [297]. In the phase 1 study of non-Hodgkin’s lymphoma NCT02953509, the anti-CD47 antibody Hu5F9-G4 combined with rituximab showed promising activity (response rate: 50%; complete response rate: 36%) [298]. Besides, more anti-CD47 antibody-involved strategies achieve encouraging results in solid and hematological malignancies [299–301].

Besides the CD47-SIRPα axis, other immune checkpoints, such as Siglec receptors, are also vital targets for cancer immunotherapy [302]. Similar to PD-1 signaling, sialoglycan ligands bind to inhibitory Siglec receptors (e.g., Siglec-7 and Siglec-9), suppressing intracellular immune signaling by recruiting SHP1/2 phosphatases [303]. Innate immune cells, especially TAMs, highly express Siglec receptors [304]. In various cancers, tumor-derived ligands (e.g., CD24 and sialoglycans) induce monocyte differentiation toward protumor TAM phenotype by Siglec-7, Siglec-9, Siglec-10, Siglec-15, and Siglec-E [287, 305–311]. Actually, Siglec signaling undermines the functions of multiple immune cells, including but not limited to DCs, NK cells, and T cells. Degenerating sialic acid residues by sialidase improves lymphocyte phagocytosis [312]. Preclinical studies have demonstrated that Siglec-15 blockade boosts antitumor immunity and inhibits tumor growth [310, 313]. Interrupting CD24-Siglec-10 interaction by anti-CD24 antibody improves phagocytic clearance of cancer cells by macrophages [287]. Moreover, other immune checkpoints and scavenger receptors are also identified as important regulators for TAM polarization and functions, such as LILRB, PD-1, and P-selectin glycoprotein ligand 1 (PSGL1) [314–319]. At present, most agents targeting these pathways are in clinical evaluation except for anti-PD-1/PD-L1 antibodies.

#### TAM metabolism regulators or other novel agents reprogramming TAM

Driven by nutrient deprivation and hypoxia, dysregulated metabolic conditions in the TME promote the accumulation of TAMs [320]. The by-product of glycolysis is lactic acid, which could promote the polarization of macrophages toward the M2-like phenotype [321]. Agents targeting glycolysis, such as 2-deoxy-D-glucose (2-DG), reverse M2 polarization [322]. Moreover, the respiratory complex I inhibitor metformin reprograms the TME: increasing immunoinhibitory CD11c^+^ but decreasing immunosupportive CD163^+^ TAMs, and strengthening macrophage phagocytosis against cancer cells [323]. Inhibiting tumor-derived retinoic acid induces the differentiation of monocytes toward immunostimulatory DCs rather than TAMs [324]. Also, glutamine metabolism inhibitors retard tumor growth by rewiring TAMs toward the M1-like phenotype [325]. Furthermore, IDO1-mediated tryptophan metabolism, tumor-derived PGE2, and oxysterol receptor LXR transcription factor also maintain the immunoinhibitory functions of TAMs [213, 326, 327]. Agents blocking these molecules have immense potential and broad prospects. Apart from regulating tumor metabolites, other novel agents, such as anti-IL-1 antibodies and nanoparticles containing mRNAs encoding IRF5-IKKβ or miRNA-155, effectively reprogram TAMs toward antitumor effectors [328–330].

#### Macrophage-based cell therapy

The TAM pool is dynamically replenished by peripheral circulating monocytes, which are constantly trafficked into the TME. Therefore, monocytes could be used as Trojan horses to delivery agents into tumors [331–333]. Nanoparticle-loaded monocytes exhibit superior antitumor activity to free nanoparticles [334]. Also, genetically engineered hematopoietic progenitors with high expression of Tie-2 and IFN-α effectively migrate to tumors and reshape the TME by releasing IFN-α [335]. Genetically engineered myeloid cells highly expressing IL-12 improve T cell response and inhibit tumor growth [336]. Furthermore, engineered particles (containing cytokines such as IFN-α) adhering to macrophage surfaces could facilitate TAMs to maintain their antitumor phenotype in the hostile TME [337].

Apart from engineering macrophages for drug delivery, macrophage engineered with CAR (CAR-M) therapy is also a promising manner to mobilize antitumor immune response [338, 339]. Similar to CAR-T cells, CAR-M contains extracellular antigen-recognizing, transmembrane, and intracellular domains. However, ZAP-70, a kinase for T cell activation, is not available in macrophages. Instead, CAR-M transduces phagocytic signals by another kinase Syk, which contains tSH2 domain and binds to CD3ζ [340]. Besides CD3ζ, other domains with immunoreceptor tyrosine-based activation motifs (ITAMs), such as multiple epidermal growth factor-like domains protein 10 (Megf10) and Fc receptor (FcRγ), also elicit phagocytosis of macrophages [341, 342]. CD3, CD147, FcR, and Megf10 are commonly utilized intracellular signaling domains in CAR-M products [343].

The first CAR-M product was developed in 2018, initially referred to as CAR-phagocytes (CAR-Ps), by employing a lentiviral vector to introduce a CAR with either Megf10 or FcRγ as the cytosolic domain into mouse macrophages [342]. These CAR-Ps displayed specific engulfment of entire human cancer cells, particularly when a tandem PI3K p85 subunit was integrated into the CAR. Although this study primarily focused on the impact of CAR on phagocytosis while excluding other essential anti-tumor functions carried out by macrophages, it marked a significant milestone in CAR-based immunotherapy [342]. Moreover, CAR-M cells possess the capacity to stimulate the transformation of M2 macrophages into M1 and release proinflammatory cytokines in the TME. It was reported that anti-HER2 CAR-M cells not only displayed tumor-killing capabilities but also induced a proinflammatory TME. Additionally, CAR-M products could enhance the activity of tumor-specific T cells by generating proinflammatory chemokines and cytokines, reprogramming M2-like into M1-like macrophages, and increasing the expression of antigen presentation machinery [341]. Furthermore, the extracellular matrix (ECM) hampers immune cell infiltration into the TME, limiting the efficacy of immunotherapy. In contrast, macrophages are naturally attracted to the TME, break down the ECM, and consequently represent the most abundant immune cell population in tumors by secreting MMPs. Shen et al. engineered CAR-M cells utilizing CD147 as the intracellular signaling domain (referred to as CAR-147 M). They observed that when these CAR-147 M cells were co-cultured with target cells, there was a significant increase in MMP expression. Although this boost in MMPs did not affect tumor cell proliferation in vitro, CAR-147 M cells rapidly accumulated at the tumor site when administered in vivo. This led to a reduction in tumor collagen deposition and promoted the infiltration of immune cells, ultimately resulting in significant tumor suppression [344]. Generally, CAR-M has some advantages over CAR-T cells in solid tumors, especially enhanced trafficking and infiltration into the TME [345, 346]. At present, most CAR-M products are at the preclinical stage, and only one autologous CAR-M targeting Her-2 is in clinical evaluation (CT-0508, NCT04660929, Phase 1) [339, 347].

In addition to Trojan horse strategies and CAR-M, there exist combinations that merge elements from both strategies. Nanocomplexes comprised of nanocarriers designed for macrophage targeting and plasmid DNA encoding CAR-interferon-γ, when administered in vivo, induce the development of CAR-M1 macrophages. These specialized macrophages exhibit the ability to engage in CAR-mediated cancer cell phagocytosis, orchestrate anti-tumor immunomodulatory responses, and effectively impede the growth of solid tumors [348].

## Harnessing MDSC for cancer therapy

MDSCs are a heterogeneous population of cells with immunosuppressive effects [349]. Under normal physiological conditions, bone marrow cells differentiate into mature subsets, including DCs, macrophages, and granulocytes (also termed terminally differentiated cells) [350]. However, the differentiation process of MDSC is disturbed by the TME, arresting it in an immature state [351]. The immunosuppressive nature of MDSCs contributes to cancer progression by promoting immune evasion and treatment resistance. For several solid tumors and hematologic malignancies, elevated levels of MDSCs have been associated with poor prognosis and treatment response [352–360]. Understanding the role of MDSCs in cancer is crucial for developing effective therapeutic strategies. Targeting MDSCs and modulating their immunosuppressive functions may hold promise in enhancing antitumor immune responses and improving patient outcomes.

### MDSCs and their protumor effects

MDSCs could be mainly classified into two cell subsets named polymorphonuclear MDSC (PMN-MDSC, similar to neutrophils in phenotype and morphology) and monocytic MDSC (M-MDSC, similar to monocytes in phenotype and morphology) [361]. PMN-MDSCs typically account for more than 80% of all MDSCs in various cancers [361]. Besides, within the overall population of MDSCs, there is a small subset comprising less than 3% of cells that possess myeloid colony-forming capability [361]. In mice, MDSCs are distributed in peripheral blood, bone marrow, spleen, lung, liver, and tumors. Murine PMN-MDSC is commonly defined as CD11b^+^Ly6G^+^Ly6C^lo^, while murine M-MDSC is defined as CD11b^+^Ly6G^−^Ly6C^hi^ [362]. In humans, MDSCs are distributed in peripheral blood and tumors. Predominantly, human PMN-MDSC is defined as CD11b^+^CD15^+^HLA-DR^lo^CD66b^+^, while human M-MDSC is defined as CD11b^+^CD14^+^CD33^+^HLA-DR^lo/−^ [363]. Moreover, Lin^−^HLA-DR^−^CD33^+^ cells (early-stage MDSC or e-MDSC) are a mixture of MDSCs containing more immature progenitors [364].

**Fig. 3:**
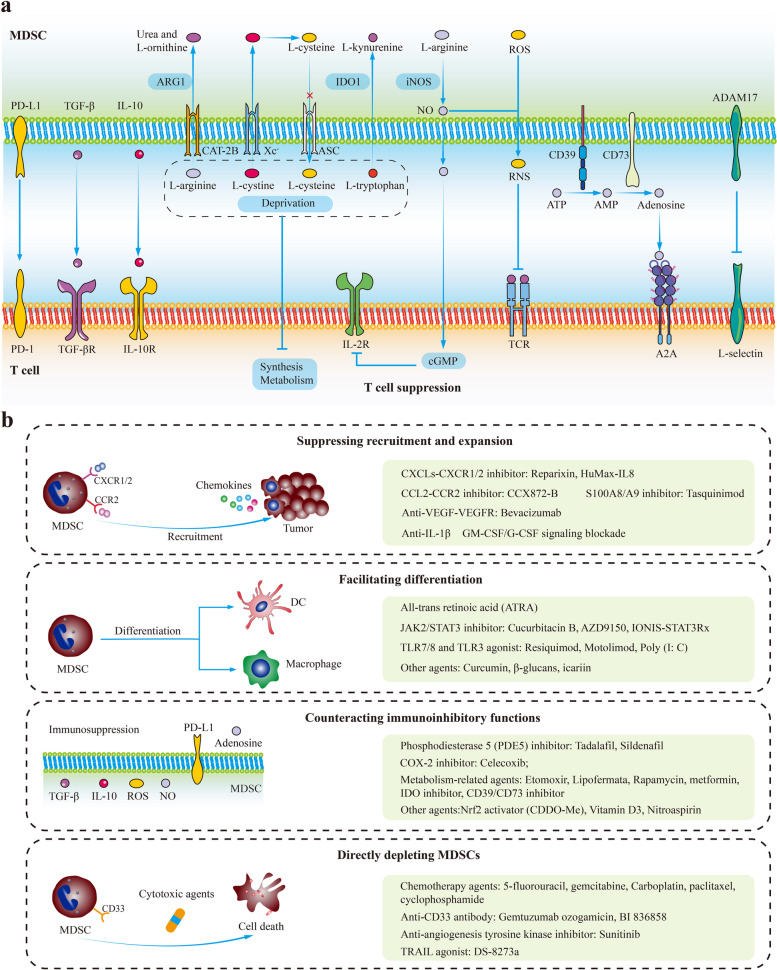
MDSC-mediated T cell suppression and MDSC-targeted therapies. **a** MDSC-mediated T cell suppression. Although MDSCs have been implicated in undermining the functions of multiple immune cells, their main targets are T cells. MDSCs cause immune suppression by upregulating TGF-β, IL-10, IDO, iNOS, ARG1, ROS, PD-L1, and depleting cystine and cysteine in the TME. Besides, the ADAM17 on MDSCs exerts immunosuppressive effects by downregulating L-selectin (T cell homing receptor) on naïve T cells. **b** MDSC-targeted therapies can be categorized into four groups: suppressing the recruitment and expansion of MDSCs; facilitating the differentiation of MDSCs into mature myeloid cells; counteracting the functions of MDSCs; and directly depleting MDSCs. Abbreviations: MDSC, myeloid-derived suppressor cell; ASC, asc­type amino acid transporter; CAT-2B, cationic amino acid transporter 2B; Xc^−^, cystine-glutamate transporter; IDO, indole-2,3 dioxygenase; NO, nitric oxide; iNOS, inducible nitric oxide synthase; TCR, T cell receptor; ROS, reactive oxygen species

The primary characteristic of MDSCs is immune suppression. Although MDSCs have been implicated in undermining the functions of multiple immune cells, their main targets are T cells. MDSCs cause immune suppression by upregulating TGF-β, IL-10, IDO, iNOS, ARG1, PEG2, reactive oxygen species (ROS), PD-L1, and depleting cystine and cysteine in the TME (Fig. 3a) [21, 365, 366]. Besides, the ADAM17 on MDSCs exerts immunosuppressive effects by downregulating L-selectin (T cell homing receptor) on naïve T cells [367, 368]. It has been confirmed that PMN-MDSCs and M-MDSCs prefer different manners to inhibit T cell response. PMN-MDSCs preferentially produce ARG1, ROS, peroxynitrite, and PGE2, while M-MDSCs preferentially generate NO, TGF-β, and IL-10 [351, 369, 370]. Apart from cytotoxic T cells, MDSCs impair other tumoricidal immune cells, including DCs, B cells, and NK cells [371–373]. Furthermore, MDSCs weaken antitumor immunity by inducing the differentiation or enhancing the functions of immunosuppressive cells such as TAMs and Tregs [374–376].

In addition to exerting immunosuppressive effects, MDSCs contribute to tumor progression by promoting tumor angiogenesis, maintaining cancer stemness, inducing EMT, and facilitating premetastatic niche formation [377]. On the one hand, MDSCs support vascularization by generating VEGF and MMP-9 [378]. On the other hand, some MDSCs have the potential to differentiate toward endothelial-like cells, directly incorporating into tumor endothelium [379]. Moreover, exosomal S100A9 released by MDSCs increases the stemness of colorectal cancer in a HIF-1α-dependent manner [380]. MDSC-endowed stemness qualities are also observed by triggering STAT3-NOTCH crosstalk and inducing miRNA-101 in breast and ovarian cancer cells [381, 382]. Besides, in murine colorectal cancer models, increased CXCL1 in premetastatic tissues attracts CXCR2^+^ MDSCs, which support cancer cell survival and promote metastatic niche formation [383].

### MDSC-targeted therapies

The significant involvement of MDSCs in tumor development has sparked the exploration of MDSC-targeted therapies. These strategies can be categorized into four groups: (1) suppressing the recruitment and expansion of MDSCs; (2) facilitating the differentiation of MDSCs into mature myeloid cells; (3) counteracting the functions of MDSCs; and (4) directly depleting MDSCs (Table 5) (Fig. 3b) [21, 384].

**Table 5:** MDSC-based therapeutic strategies

Classification	Target	Agent	Representative clinical trials	Cancer type	Phase
Suppressing the recruitment and expansion of MDSCs	CXCR1/2	Reparixin	NCT02370238	TNBC	2
		Navarixin	NCT03473925	Solid tumors	2
		SX-682	NCT04574583	Solid tumors	1/2
	CXCR2	AZD5069	NCT03177187	Prostate cancer	1/2
	CXCL8	HuMax-IL8	NCT02536469	Solid tumors	1
	S100A8/A9	Tasquinimod	NCT01234311	Prostate cancer	3
	VEGF	Bevacizumab	NCT02669173	Glioblastoma	1
	VEGFR	Pazopanib	NCT00866697	Gynecologic cancer	3
		Cabozantinib	NCT01605227	Prostate cancer	3
		Regorafenib	NCT01853319	Colorectal cancer	3
		Sorafenib	NCT01234337	TNBC	3
Facilitating the differentiation of MDSCs	Nuclear retinoid receptors	All-trans retinoic acid	NCT00617409	SCLC	2
	STAT3	AZD9150	NCT03421353	NSCLC	1
		IONIS-STAT3Rx	NCT01563302	Solid tumors and lymphoma	1/2
	TLR9	CpG ODN	NCT04952272	Solid tumors	1
	TLR7/8	Resiquimod	NCT00821652	Solid tumors	1
	TLR7/8	Motolimod	NCT02431559	Ovarian cancer	1/2
	TLR3	NS-9 Poly (I:C)	NCT00094003	Solid tumors with liver metastases	1
Suppressing the functions of MDSCs	COX-2	Celecoxib	NCT03026140	Colon cancer	2
	PDE5	Tadalafil	NCT03993353	Head and neck cancer	2
		Sildenafil	NCT00752115	NSCLC	2/3
	HDAC1/3	Entinostat	NCT02708680	TNBC	1/2
	HDAC6	Ricolinostat	NCT02091063	Lymphoma	1/2
	Nrf2	CDDO-Me	NCT00529438	Lymphoma	1
	COX-1	Nitroaspirin	NCT00331786	Colon cancer	1
	mTOR	Everolimus	NCT04203901	Renal cell carcinoma	2
	Glycolysis	Metformin	NCT03709147	Lung cancer	2
	IDO	Indoximod	NCT01792050	Breast cancer	2
	CD73	MEDI9447	NCT02503774	Solid tumors	1
Directly depleting MDSCs	CD33	Gemtuzumab ozogamicin	NCT03531918	Acute myeloid leukemia	1/2
		BI 836858	NCT01690624	Acute myeloid leukemia	1
	Cytotoxic drugs	5-Fluorouracil	NCT03299660	Rectal cancer	2
		Gemcitabine	NCT03302247	NSCLC	2
		Carboplatin	NCT05841472	NSCLC	2
		Paclitaxel	NCT04815408	Ovarian cancer	2
		Capecitabine	NCT03111732	Biliary tract carcinoma	2

*MDSC* myeloid-derived suppressor cell, *SCLC* small cell lung cancer, *NSCLC* non-small cell lung cancer, *TNBC* triple-negative breast cancer, *TLR* toll-like receptor, *VEGF* vascular endothelial growth factor, *IDO* indoleamine 2,3-dioxygenase, *PDE5* phosphodiesterase 5, *HDAC* histone deacetylase, *COX2* cyclooxygenase-2

#### Suppressing the recruitment and expansion of MDSCs

MDSCs migrate to tumors under the guidance of chemokine pathways such as CXCLs-CXCR1/2 and CCL2-CCR2 [385, 386]. CXCLs-CXCR1/2 blockade improves the antitumor activities of immunotherapies in various murine models by preventing the trafficking of PMN-MDSCs into the TME [387–389]. So far, CXCR1/2 inhibitors (e.g., AZD5069, Reparixin, Navarixin, and SX-682) and anti-CXCL8 antibodies neutralizing IL-8 (also termed CXCL8 in humans) (e.g., HuMax-IL8 and ABX-IL8) are undergoing clinical evaluation [390, 391]. Besides, IL-1β contributes to the recruitment and expansion of MDSCs and modulates their immunoinhibitory functions in the TME [392]. Inhibiting IL-1β or NLRP3 inflammasome (a key component for IL-1β maturation) reduces MDSCs and enhances antitumor immunity in head and neck squamous cell carcinoma models [393–397]. Additionally, GM-CSF leads to MDSC accumulation and weakens cancer antigen-specific T-cell response [398]. At the same time, G-CSF initiates MDSC mobilization and promotes tumor angiogenesis [399]. GM-CSF/G-CSF blockade with antibodies reduces MDSC accumulation and overcomes cancer immune escape [400, 401]. Moreover, MDSCs simultaneously express S100A8/A9 and their receptors RAGE, forming a positive feedback loop that promotes the recruitment of MDSCs and amplifies their immunosuppressive capabilities. S100A8/A9 inhibitors disturb this positive feedback loop, diminish MDSC accumulation, and retard tumor growth in various murine models [402–404]. Furthermore, anti-VEGF-VEGFR therapies also inhibit MDSC recruitment by blocking VEGFR1 signaling of MDSCs [405, 406].

#### Facilitating the differentiation of MDSCs into mature myeloid cells

All-trans retinoic acid (ATRA) regulates cell differentiation, proliferation, and apoptosis by nuclear retinoid receptors [407]. Differentiation therapy with ATRA has altered the therapeutic paradigm of acute promyelocytic leukemia and significantly improved patient outcomes [408]. Similarly, ATRA could promote the differentiation of immature MDSCs toward terminated differentiated myeloid cells (DCs, macrophages, and granulocytes) [409]. In patients with metastatic renal cell carcinoma, ATRA treatment substantially reduces MDSC in peripheral blood, increases the cDC/pDC ratio, and enhances antigen presentation and antigen-specific T-cell response [410]. In multiple clinical trials of lung cancer (NCT00617409) and melanoma (NCT02403778), additional ATRA treatment significantly augments immunotherapy and chemotherapy [411–413]. Moreover, constitutive STAT3 activation prevents the differentiation of immature myeloid cells and maintains their immunosuppressive properties [414, 415]. In patients with advanced lung cancers, Cucurbitacin B (JAK2/STAT3 inhibitor) decreases the ratio of immature-to-mature myeloid cells in peripheral blood [416]. In patients with diffuse large B-cell lymphomas, AZD9150 (antisense oligonucleotide of STAT3) reduces peripheral PMN-MDSCs as well [417]. The synergistic effects between STAT3 inhibitors and immunotherapies have been validated in a series of preclinical and clinical studies [418–422].

TLRs also play an important role in the maturation and differentiation of MDSCs. CpG oligodeoxynucleotides (termed CpG ODN, TLR9 agonist) stimulates antitumor immunity by activating CD8^+^T/NK cells, inducing the differentiation of M-MDSC toward M1-like macrophages [423–425]. In vivo experiments demonstrate that CpG effectively promotes the maturation of MDSC and abrogates MDSC-mediated T-cell suppression by triggering IFN-α production of pDCs [426]. Also, TLR7/8 and TLR3 agonists, such as resiquimod, motolimod, and Poly (I: C), relieve MDSC-induced immune evasion and revive antitumor immune response [114, 427–429]. Furthermore, some novel agents, such as curcumin, β-glucans, and icariin, drive the differentiation of MDSCs into DCs and macrophages and undermine their suppressive functions [430–432].

#### Counteracting the functions of MDSC

The COX-2-PGE2 axis is the key pathway to maintain the immunosuppressive functions of MDSCs [433–435]. On the one hand, PGE2 in the TME attracts MDSCs by CXCL12-CXCR4 [436]. On the other hand, PGE2 from tumor cells triggers the nuclear p50/NF-κB signaling in M-MDSCs, which reprograms their response to IFN-γ and decreases TNF-α generation [437]. Besides, paracrine PGE2 induces MDSCs to upregulate COX-2 expression, which could stimulate autocrine PGE2 production, forming a positive feedback loop [438]. This PGE2-COX-2 positive feedback loop facilitates to stabilize MDSC phenotype [438]. Agents targeting COX-2-PGE2 signaling hamper the immunoinhibitory functions of MDSCs and improve the sensitivity to immunotherapies [439, 440]. For example, celecoxib (COX-2 inhibitor) decreases the production of ROS and NO in MDSCs and reverses T-cell tolerance [441]. Besides, celecoxib combined with CD40 agonist therapy effectively increases CXCL10 but reduces ARG1 in MDSCs. As a result, antitumor immunity is restored, and tumor growth is suppressed in glioma-bearing mice [442].

Additionally, phosphodiesterase 5 (PDE5) inhibitors such as tadalafil and sildenafil reduce the levels of ARG1, iNOS, and IL-4Rα (myeloid suppressor cell suppressive marker) [443, 444]. In clinical studies of melanoma (EudraCT: 2011–003273-28) and head and neck squamous cell carcinoma (NCT00843635 and NCT00894413), tadalafil reduces MDSC frequency, hampers the immunoinhibitory properties of MDSCs, and augments cancer-specific immunity [445–447]. Moreover, epigenetic regulators such as histone deacetylase inhibitors (HDACis) have substantial influences on the functions of MDSCs. In murine tumor models, HDACi treatment significantly downregulates the expression of COX-2, ARG1, and iNOS in MDSCs [448, 449]. The class I HDACi entinostat mainly modulates the functions of PMN-MDSCs, while class II HDAC6 inhibitor ricolinostat primarily regulates the functions of M-MDSCs [450]. Moreover, other novel agents such as Nrf2 activator (CDDO-Me), vitamin D3, and nitroaspirin (the derivative of aspirin with nitro moiety) are identified as negative regulators for MDSC-mediated immunosuppression [451–453].

The functions of MDSCs could be suppressed by disturbing their metabolism. Due to the high consumption and active fatty acid oxidation (FAO) of MDSC, inhibiting some key molecules in FAO impedes MDSC-mediated immune suppression [454]. Agents targeting FAO rate-limiting enzymes such as etomoxir (targeting enzyme CPT1) and lipofermata (targeting enzyme FATP2) remarkably abrogate the immunosuppressive activities of MDSCs in the TME [455, 456]. In addition to fatty acid metabolism, glycolysis also has positive effects on the survival and activity of MDSCs. In murine tumor models, tumor-infiltrating MDSCs have more active glycolysis and mTOR signaling [457]. Rapamycin (mTOR inhibitor) downregulates the quantity and activity of M-MDSCs in mice [458]. Also, a glycolysis modulator (metformin) counteracts the inhibitory functions of MDSCs by impeding the expression and enzymatic activity of CD39/CD73 [459]. Furthermore, targeting other metabolic enzymes or metabolites such as IDO (converting tryptophan to kynurenine) inhibitors and CD39/CD73 (converting ATP to adenosine) inhibitors also reprograms MDSCs and contributes to the renaissance of antitumor response [384, 460, 461].

It is important to note that certain agents affecting metabolism can also impact immune cells within the TME apart from MDSCs. For instance, the activation of STAT3 signaling leads to a metabolism biased toward FAO in CD8^+^ T cells, which impairs their functionality and contributes to the development of obesity-related breast cancer. On the other hand, inhibiting FAO enhances the performance of CD8^+^ T effector cells and inhibits tumor growth [462]. Additionally, the peroxisome proliferator-activated receptor agonist Bezafibrate stimulates mitochondria, enhancing oxidative phosphorylation, glycolysis, and FAO, ultimately leading to improved functionality in T cells infiltrating tumors [463]. Furthermore, the costimulatory signal 4-1BB enhances the glucose and fatty acid metabolism in T cells to meet their growing energy demands. The effects on the T cell cycle and anti-apoptotic activity mediated by 4-1BB signaling are entirely nullified by the FAO inhibitor etomoxir [464]. Moreover, there is evidence that Metformin therapy restores the impaired metabolic function of hepatic CD8^+^ T cells in non-alcoholic steatohepatitis (NASH) and enhances the efficacy of anti-PD-1 treatment in liver tumors associated with NASH [465]. Furthermore, the impact of immunometabolism on other immune cells, such as DCs and macrophages, has been confirmed. The anabolic and catabolic processes substantially influence the immunogenicity and tolerogenicity of DCs, while succinate and citrate directly regulate macrophage functions [466]. Hence, it is essential to comprehensively consider the effects of metabolism-modulating agents on various components of the TME beyond MDSCs to achieve optimal immunotherapy efficacy.

#### Directly depleting MDSCs

Some chemotherapeutic agents could selectively eradicate regulatory immune cells, especially MDSC, and alleviate immune suppression [467]. For example, 5-fluorouracil and gemcitabine induce the MDSC apoptosis and restore tumor-specific CD8^+^ T cell response [468]. Carboplatin and paclitaxel cause MDSC depletion and boost therapeutic vaccination-mediated immune response [469]. Besides, low-dose capecitabine reduces circulating MDSCs and increases cytotoxic immune infiltration in the TME [470]. It is notable that some cytotoxic agents might also have positive effects on MDSCs, such as cyclophosphamide (CTX). The difference could be attributed to agents, administration schedules and doses, and heterogeneity of sampling [471]. Generally, these agents are not MDSC-specific, with cytotoxic effects on all rapidly proliferating, even lymphocytes in the TME. Relatively, therapies targeting CD33 have better specificity for MDSCs [472]. Fc-engineered anti-CD33 antibodies (BI 836858) and anti-CD33 antibody-conjugated drug (gemtuzumab ozogamicin) could specifically eliminate MDSCs [472, 473]. Additionally, agonists of TNF-related apoptosis-induced ligand (TRAIL) receptors and anti-angiogenesis tyrosine kinase inhibitor sunitinib are regarded as MDSC eliminators as well [474, 475].

Collectively, MDSCs play a crucial role in tumor development, leading to the exploration of four main categories of MDSC-targeted therapies. These approaches include (1) suppressing MDSC recruitment and expansion through blockade of chemokine pathways and cytokines, (2) promoting MDSC differentiation into mature myeloid cells using agents like ATRA, STAT3 inhibitors, and TLR agonists, (3) countering MDSC functions by targeting the COX-2-PGE2 axis and metabolic pathways, and (4) directly depleting MDSCs, often through chemotherapeutic agents like 5-fluorouracil and gemcitabine or specific MDSC-targeting therapies like anti-CD33 antibodies. Notably, some metabolic modulators can affect other immune cells in the TME. These strategies offer potential in enhancing cancer immunotherapy by either reducing MDSC numbers or neutralizing their suppressive functions, but their broader effects on immune cells need to be considered for optimal outcomes.

## Targeting NK for cancer immunotherapy

NK cells are a type of immune cell that make up the innate lymphoid cellular defense and surveillance system [476, 477]. When encountering tumor cells, it serves as the primary sentinel in safeguarding organismal health. In humans, NK cells lack membranal TCR and CD3 molecules but have neural cell adhesion molecule (NCAM, also known as CD56), along with activating and inhibitory receptors [478, 479]. Particularly, unlike other surface biomarkers only found in the bloodstream, CD335 is an activating receptor that can also identify NK cells in formalin-fixed paraffin-embedded tissue specimens [480]. Commonly, NK cells take up approximately 5%-20% of circulating lymphocytes in humans [481]. NK cells can be activated and exert cytotoxic effects independent of specific antigen recognition, as they recognize foreign organisms and malignancies through the aforementioned stimulatory and inhibitory receptors [476].

### The biology of human NK cells

It is well established that human NK cells proceed through five discrete stages in lineage derivation and development [482]. Human NK cells, together with other kinds of innate lymphoid cells (ILCs), are derived from multipotent CD34^+^ hematopoietic progenitors [483, 484]. A subset of these progenitors is committed into α-lymphoid precursor (αLP) cells by expressing integrin α4β7 [485, 486]. Subsequently, αLP cells expressing CXCR6 are able to develop into precursor NK and ILC-3 subtypes [487]. The symbolic event of precursor NK occurrence is the IL-1R1 expression on αLP cells [488, 489]. Predominant precursor NK cells undergo further development and maturation in bone marrow, while a minority of cells undergo maturation in peripheral lymphoid organs [490, 491]. In these sites, NK cells gradually express specific surface receptors driven by multiple transcription factors, including T-bet, Id2, E4BP4, and Eomesodermin (Eomes) [492–495]. In brief, NK cell development involves dynamic changes in lineage-specific biomarkers, with a gradual decrease in progenitor and precursor markers and an increase in bioactive receptors.

The trafficking, homing, and activation of NK cells are mutually reinforcing processes that complement the maturation of these cells. Specifically, immature NK cells require certain factors to facilitate their trafficking during maturation. Most NK cells undergo maturation within a specialized niche located in bone marrow, where they are surrounded and nourished by parenchymal sinusoidal vessels [496]. Only after CXCR4 is downregulated, NK cells migrate from bone marrow into the sinusoidal vessel and subsequently into peripheral blood [497]. Additionally, various chemokines and integrins, along with their corresponding receptors and ligands, are involved in this process. Therefore, distinct types of chemokines and integrins can be identified as biomarkers for circulating or tissue-resident NK cells [498, 499]. The migration of NK cells from sinusoidal vessels into circulation also needs the participation of various factors, particularly CX3CR1, S1P5, and CXCR6 [500–505]. Additionally, CX3CR1 boosts the infiltration of NK cells into the central nervous system, which is traditionally considered the forbidden zone for immune cells [504, 506].

The mature NK cells can be further classified into two major subtypes: CD56^bright^CD16^dim/−^ and CD56^dim^CD16^+^ [490], while rare cells differentiate into memory NK cells under specific stimuli [507, 508]. The CD56^bright^CD16^dim/−^ subset possesses poor cytotoxicity and minor circulating proportion, which could further differentiate into CD56^dim^CD16^+^ cells [481]. The CD56^bright^CD16^dim/−^ subset is more commonly observed in lymph nodes, the gastrointestinal tract, and tonsils, where the overall proportion of NK cells is lower [478]. In these sites, they exert more secretory biologic function rather than cell lysed function [509–511]. On the contrary, the CD56^dim^CD16^+^ subset is regarded as cytotoxic NK cells, which could directly eradicate tumor cells by death receptor signaling or cytotoxic effector molecules [512].

After licensing, NK cells are directly activated, equipped with a diverse array of inhibitory and activating surface receptors, independent of MHC-restricted antigen recognition when encountering detrimental factors [492]. In the TME, NK cells are activated by the construction changes or expression downregulation of MHC-I molecules [513]. Also, NK cells can be activated by stimulatory receptors such as NKp30, NKp44, and NKp46 [514]. Additionally, the CD16 receptor on NK cells exerts separate activating functions after being engaged by the immunoglobulin-opsonized cells. This process elicits the phosphorylation of the ITAM domain of FcεRIγ and CD3ζ on the surface of NK cells, ultimately culminating in ADCC [515, 516].

### The roles of NK cells in antitumor immunity

However, the antitumor activity of NK cells is limited by multiple factors, such as insufficient NK cell infiltration and the hostile TME [517, 518]. It has been validated that cancer-derived exosomes and hypoxia could blunt NK cell activity [519, 520]. Besides, some modulatory immune cells and cytokines, such as TGF-β, activin-A, and adenosine, also contribute to the immunosuppression of tumor-infiltrating NK cells [521–525].

**Fig. 4:**
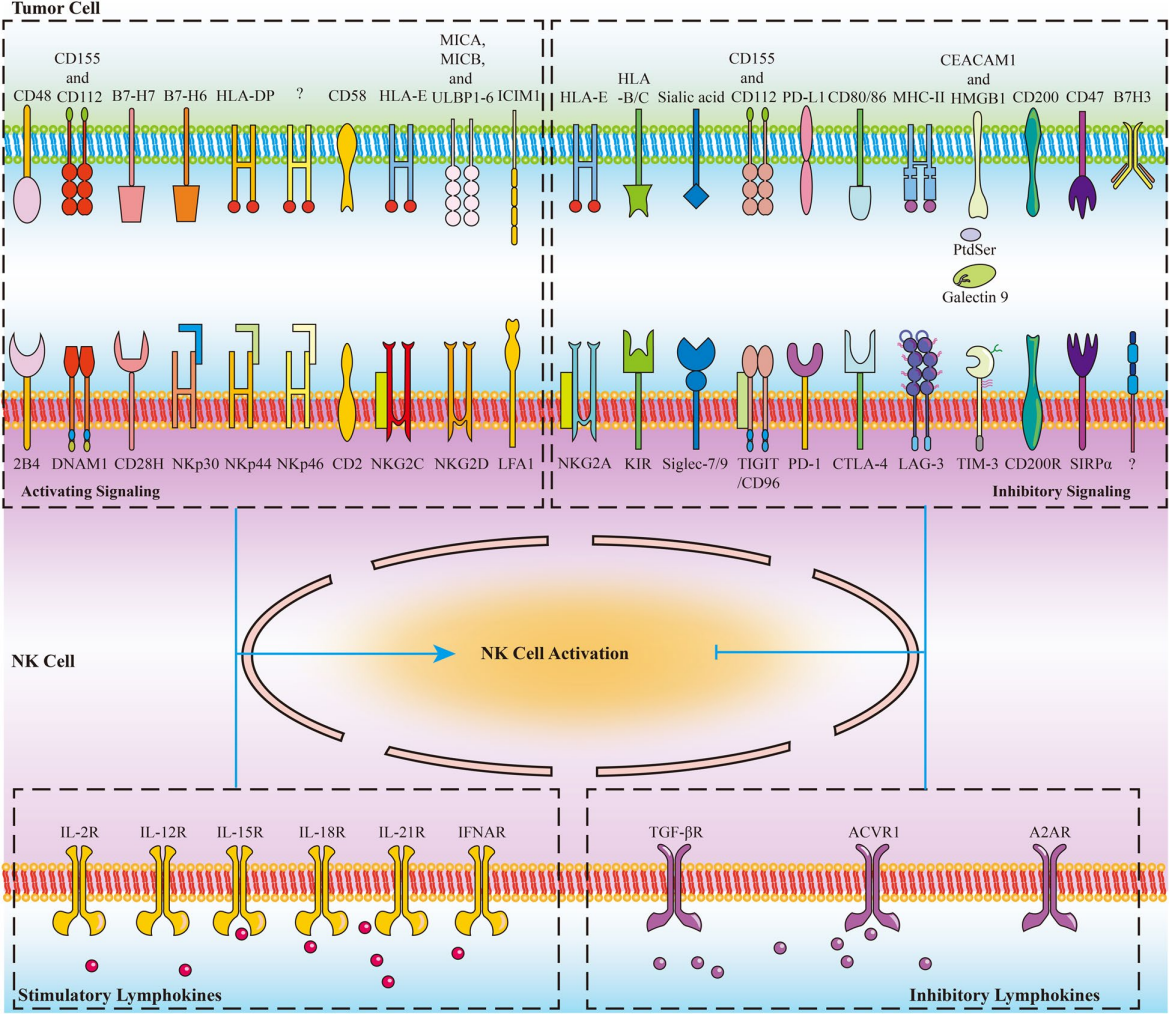
Interaction between NK cell and the TME. Schematic diagram depicting primary receptors expressed by NK cells and their corresponding ligands on tumor cells or cytokines in the TME. Activating stimulative receptors triggers an intracellular signaling cascade that activates NK cells and vice versa. The two factors dynamically modulate the behavioral pattern of NK cells, whose disequilibrium may lead to immune evasion or clearance. Abbreviations: NK cell, natural killer cell; TME, tumor microenvironment; Ecto-CRT, ecto-calreticulin; A2AR, A2a adenosine receptor; ACVR1, activin receptor type 1

High NK cell abundance in the TME predicts a favorable prognosis in a myriad of cancers [241, 526–528], and NK cells suppress tumorigenesis by executing immunosurveillance [529, 530]. In the TME, NK cell activation is determined by activating and inhibiting signals, such as NKG2D, 2B4, DNAM1, LFA1, CD28H, IL-12, IFN-α and TGF-β (Fig. 4) [531–533]. Notably, HLA-E exerts dualistic immunoregulatory effects on NK cells when binding to different receptors [534–538]. Activated NK cells can eliminate tumor cells by releasing perforin and granzymes, as well as by inducing apoptosis via ADCC, FasL, or TRAIL [531, 539]. Besides, NK cells secrete cytokines, including IFN-γ and TNF-α, which lead to tumor growth arrest [540]. The ruptured tumor cells will unleash neoantigens, subsequently prompting the adaptive immune response [541]. As the communicative bridge between innate and adaptive immunity, DC plays crucial and intricate roles in antitumor immune responses. Additionally, NK cells promote the recruitment of cDCs into the TME [57]. A novel type of NK cells, termed induced pluripotent stem cells (iPSCs)-derived NK cells, is reported to recruit T cells into the TME and augment the therapeutic effect of immune checkpoint inhibitors [542]. Apart from executing immune surveillance and elimination functions by tissue-resident NK cells, circulating NK cells can also prevent tumor metastasis by activating the NKp46/NCR1 signaling [478, 543]. The mechanisms underlying the recognition of tumor cells by NK cells highlight the perspectives for NK cell-targeted strategies, particularly in cold tumors lacking neoantigens.

### Developing NK cell-targeted therapies

**Fig. 5:**
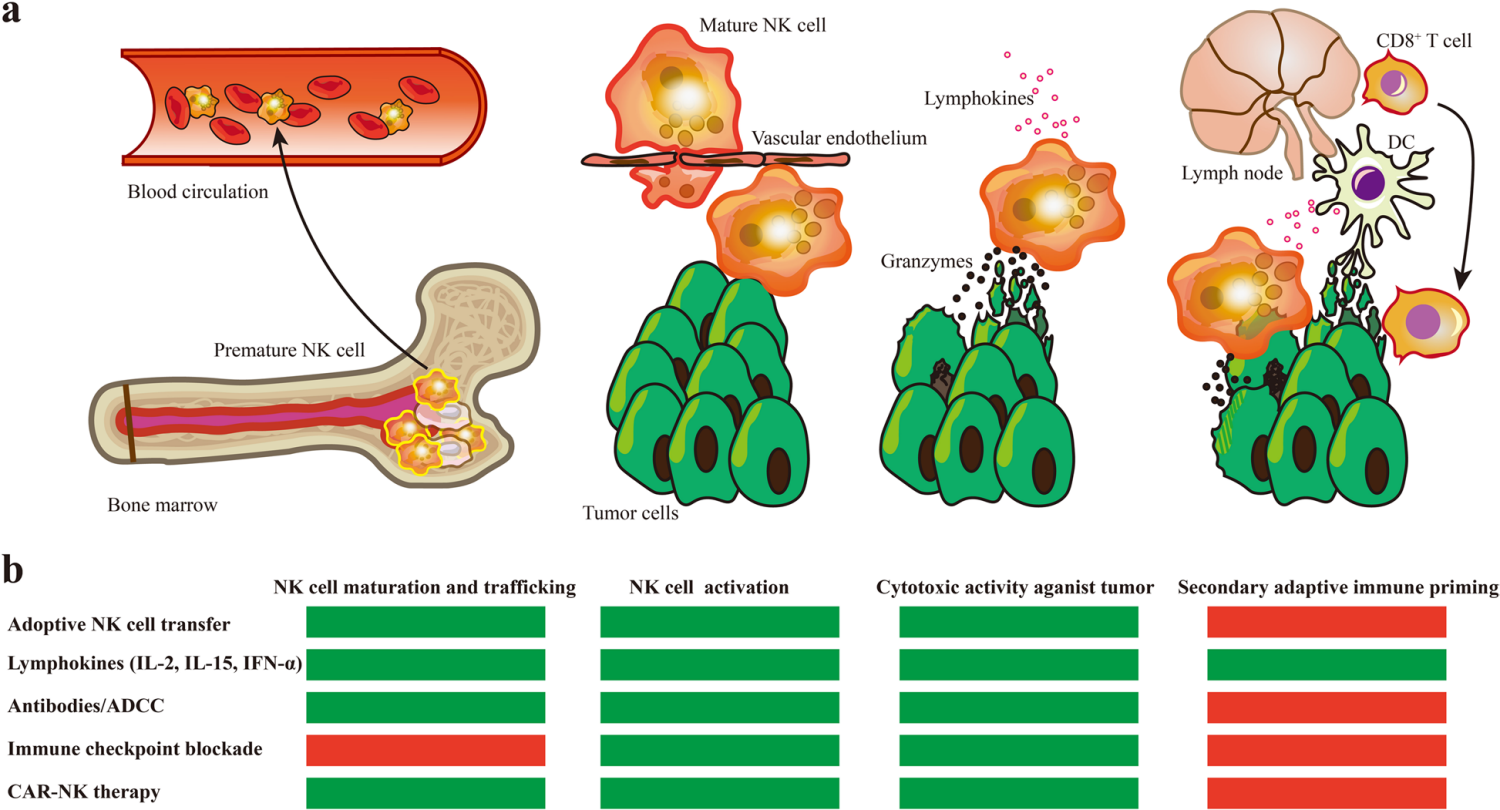
NK cell-based therapeutic strategies. **a** NK cell-based therapeutic strategies could enhance multiple biological processes, including trafficking, activation, tumor-killing activity, and NK cell-mediated secondary adaptive immune priming. **b** NK cell-based therapeutic strategies. The green box denotes the biological process targeted by the corresponding strategy, while the red box denotes the biological process not involved in the corresponding strategy. Abbreviations: ADCC, natural killer cell-mediated antibody-dependent cellular cytotoxicity; CAR, chimeric antigen receptor

The antitumor activity of NK cells has been unequivocally demonstrated through in vitro experiments and animal models, providing a solid rationale for investigating their potential as anticancer agents [544–546]. Generally, NK cell-based therapeutic strategies can be categorized into five distinct groups based on the sequential processes of NK cells, including trafficking, activation, effector function execution, and secondary adaptive immune priming, which synergistically reinforce each other (Fig. 5).

#### NK cell adoptive transfer

The most primitive conception of the employment of NK cells for cancer treatment is the transfer of healthy allogenous NK cells in patients with malignancies. In the 1980s, NK cell adoptive transfer technology was used to conquer hematological malignancies [544]. However, the antitumor activity of autogenous natural NK cells is moderate. To overcome the limitations of natural NK cells, engineered NK cells are developed by augmenting stimulating receptors and dampening inhibitory receptors [547, 548]. The innovation has revitalized the application of adoptive NK cell transfer. Since then, it has become a dominant therapeutic strategy [549–551], with superior safety relative to other adoptive cellular transfer therapies [552]. Despite the rosy perspective, more efforts are needed to improve NK cell infiltration and tumor specificity [553]. Furthermore, autologous activated and expanded natural killer cells, referred to as NKAE, offer a highly effective and low-toxicity strategy for multiple myeloma. In a phase 1 clinical trial, two out of five patients achieved a clinical objective response after receiving two infusions of NKAE [554]. The growing body of preclinical and clinical research in multiple myeloma has positioned NK adoptive cell therapy as a comparable treatment approach to CAR-T [551, 555, 556].

#### NK cell-stimulating lymphokine regimen

The cytokine regimen, which could enhance the cytotoxic activity of killer cells, was originally clinically used for renal cell carcinoma [557]. In this clinical study, NK cells became the predominant lymphocyte subset of peripheral blood mononuclear cells (PBMC) after the IL-2 combined IFN-γ treatment, indicating the promising application perspective of NK cell-stimulating lymphokine strategies [557]. Further investigations have been conducted to explore the efficacies of NK cell-stimulating cytokines, particularly IL-15, IL-2, and IFN-α [558–565]. These lymphokines enhance the tumor-killing activity of NK cells. Administrating exogenous cytokines might be a promising complement to other NK cell-based therapies.

#### Harnessing ADCC of NK cells

Antibodies targeting molecules on the surface of NK cells were developed at the end of the last century [566]. The classical antitumor agents, such as trastuzumab, cetuximab, and rituximab, are capable of eliciting the ADCC of NK cells [540]. Mechanically, these antibodies act as a physical bridge, linking the NK and tumor cells. For example, trastuzumab binds to CD16 on NK cells via its immunoglobulin G1 (IgG1) Fc portion and binds to HER2 on tumor cells via its Fab portion, mediating ADCC either synchronously or subsequently [567–569]. Besides, some immune checkpoint inhibitors like avelumab could trigger ADCC as well [570]. For tumor cells with high PD-L1 expression, avelumab directly guides NK cells to execute immune clearance, independent of the PD-1/PD-L1 signaling [570].

#### Immune checkpoint blockade of NK cells

Moreover, immune checkpoint inhibitors targeting NK cells, which could boost the activation and cytotoxic functions of NK cells, have emerged as a promising approach in cancer immunotherapy. Several receptors on NK cells have been recognized as immune checkpoints, including NKG2A/CD94, KIR family, LIR1, TIGIT/CD96, B7H3, PD-1, CTLA-4, LAG-3, TIM-3, CD200R, and SIRPα [532, 571]. The anti-inhibitory KIR antibody IPH2101 (1-7F9) effectively triggers NK cell-mediated killing of multiple myeloma in murine tumor models [572, 573]. Besides, anti-NKG2A antibodies could simultaneously enhance the cytotoxicity of NK and T cells against tumor cells [538, 574–576]. A recent clinical trial indicated that monalizumab (anti-NKG2A antibody) or oleclumab (anti-CD73 antibody, inhibiting adenosine production) synergized with PD-L1 blockade in advanced NSCLC patients [577]. Also, blocking TIGIT/CD96 can prevent NK cell exhaustion and trigger a potent NK cell-dependent tumor-specific T cell response [578]. Two early-stage clinical trials utilizing anti-TIGIT antibodies (vibostolimab or etigilimab), either as a monotherapy or in combination with the anti-PD-1 antibodies, presented encouraging activities in refractory solid tumors [579, 580]. Furthermore, another anti-TIGIT monoclonal antibody (tiragolumab) combined with anti-PD-L1 antibody (atezolizumab) showed a significant advantage over atezolizumab monotherapy in progression-free survival (5.4 vs. 3.6 months, HR = 0.57, *P* = 0.015) [581]. Moreover, the poor prognosis associated with B7H3 overexpression in multiple types of cancers, coupled with enhanced functions of NK cells resulting from B7H3 inhibition, is evidential for targeting B7H3 to enhance NK cell-mediated immune protection [582, 583]. ChT-1A5, a human-mouse chimeric monoclonal antibody of B7H3, can effectively trigger ADCC of NK cells against leukemic cells while sparing normal hematopoietic cells. Other immune checkpoints, such as PD-1, CTLA-4, LAG-3 [584], TIM-3 [585], CD200R [586], and SIRPα [587], are predominantly expressed in other immune cells and will not be expounded upon within this section. The major corresponding checkpoint inhibitors associated with NK cells are presented in Table 6.

**Table 6:** Completed or undergoing clinical trials on inhibitors of several predominant NK cell-associated checkpoints

Targets	Agents	Cancer types	Phase	NCT number	Status
NKG2A	Monalizumab	Hematological or solid tumors	2	NCT04333914	Completed
		LA-HNSCC	2	NCT03410030	Not yet recruiting
	S095029	Solid tumors	1	NCT05162755	Recruiting
	HY-0102	Solid tumors	1	NCT04914351	Active, not recruiting
KIR	IPH2101	MM	1	NCT01217203	Completed
			2	NCT00999830	Completed
			2	NCT01222286	Completed
		AML	1	NCT01256073	Completed
	Lirilumab	Solid tumors	1/2	NCT01714739	Completed
		Hematological malignancy	2	NCT02481297	Completed
	IPH4102	T Cell Lymphoma	2	NCT03902184	Recruiting
LIR1	AGEN1571	Solid tumors	1	NCT05377528	Recruiting
TIGIT	Belrestotug	Solid tumors	2	NCT03739710	Recruiting
			1/2	NCT05060432	Recruiting
		MM	1/2	NCT05289492	Recruiting
	BMS-986207	MM	1/2	NCT04150965	Recruiting
		Solid tumors	1/2	NCT04570839	Active, not recruiting
	Vibostolimab	Melanoma	1/2	NCT04303169	Recruiting
			1/2	NCT04305041	Recruiting
			1/2	NCT04305054	Recruiting
			3	NCT05665595	Recruiting
	Domvanalimab	NSCLC	3	NCT04736173	Recruiting
			2	NCT04791839	Recruiting
			2	NCT05676931	Recruiting
		Melanoma	2	NCT05130177	Recruiting
		Gastrointestinal tract carcinoma	2	NCT05329766	Recruiting
		Upper gastrointestinal tract adenocarcinoma	3	NCT05568095	Recruiting
		NSCLC	2	NCT04262856	Active, not recruiting
	M6223	Urothelial carcinoma	2	NCT05327530	Recruiting
	CHS-006	Solid tumors	1/2	NCT05757492	Recruiting
	Tiragolumab	Solid tumors	2	NCT03708224	Recruiting
		NSCLC	3	NCT04294810	Recruiting
		ESCC	3	NCT04543617	Recruiting
		Rectal cancer	2	NCT05009069	Recruiting
		Renal cell carcinoma	2	NCT05805501	Recruiting
		NSCLC	2	NCT03563716	Active, not recruiting
		SCLC	3	NCT04256421	Active, not recruiting
		Gastric cancer	2	NCT04933227	Active, not recruiting
			1/2	NCT05251948	Active, not recruiting
	Ociperlimab	ESCC	2	NCT04732494	Recruiting
		NSCLC	3	NCT04746924	Recruiting
			3	NCT04866017	Recruiting
			2	NCT05014815	Recruiting
		Biliary tract carcinoma	2	NCT05023109	Recruiting
		Cervical cancer	2	NCT04693234	Active, not recruiting
		Limited-stage SCLC	2	NCT04952597	Active, not recruiting
	SEA-TGT	NSCLC	2	NCT04585815	Active, not recruiting
	Etigilimab	Solid tumors	1/2	NCT04761198	Active, not recruiting
		Ovarian, primary peritoneal, or fallopian tube cancer	2	NCT05026606	Active, not recruiting
	AZD2936	NSCLC	2	NCT04995523	Recruiting
		Gastric cancer	2	NCT05702229	Recruiting
	HLX301	Solid tumors	1/2	NCT05102214	Recruiting
		Lymphoma or solid tumors	1/2	NCT05390528	Recruiting
	HB0036	Solid tumors	1/2	NCT05417321	Recruiting
B7H3	Omburtamab	CNS tumors	1	NCT01502917	Completed
		Peritoneal cancer	2	NCT04022213	Recruiting
		CNS tumors	2/3	NCT03275402	Active, not recruiting
			2	NCT04743661	Active, not recruiting
	Ifinatamab deruxtecan	Solid tumors	1/2	NCT04145622	Recruiting
		Extensive-stage SCLC	2	NCT05280470	Active, not recruiting
	Vobramitamab duocarmazine	Prostatic cancer	2/3	NCT05551117	Recruiting
	Enoblituzumab	Prostate cancer	2	NCT02923180	Active, not recruiting

*LA-HNSCC* Locoregionally advanced head and neck squamous cell carcinoma, *AML* Acute myeloid leukemia, *MM* Multiple myeloma, *NSCLC* Non-small cell lung cancer, *ESCC* Esophageal squamous cell carcinoma, *SCLC* Small cell lung cancer, *CNS* Central nervous system

#### CAR-NK therapy

Theoretically, chimeric antigen receptor-engineered natural killer cells (CAR-NK) technology represents the latest generation of NK cell adoptive cellular transfer (ADT) [541, 588, 589]. As mentioned earlier, allogeneic NK cell ADT provides notable safety advantages over allogeneic T cell ADT treatment in terms of minimizing the risk of developing graft-versus-host disease (GVHD) or a cytokine storm and neurotoxicity [590–592]. The off-the-shelf CAR-NK products, readily available for preparation in advance, hold immense potential in the battle against cancer [541]. CAR-NK cells can be prepared based on a diverse array of donor cells, including the NK-92 cell line, PBMCs, umbilical cord blood (UCB), hematopoietic progenitor cells (HPCs), and iPSCs [593]. Among these various options, the irradiated NK-92 cell line is the most commonly employed in clinical trials due to its characteristics of immortality, rapid proliferation rate, and commercial availability [594, 595]. In addition to the diverse origins of cell components, CAR-NK could be constructed to target different cancer-specific antigens, such as CD19, CD5, CD123, GFR, GD2, and Mesothelin [592]. Engineering CAR-NK cells commonly depends on viral vehicles [596–601]. Besides, exogenous CAR fragments are introduced by electroporation and liposome, with lower genetic toxicity and shorter initiation time for gene expression [601]. Afterward, the reformative transposon system is developed and exploited in clinical trials, which possesses increased safety, decreased expenditure, and enhanced editable flexibility [602, 603].

Notably, NKG2D plays a crucial role in the detection and elimination of cancer cells [604]. Typically, therapeutic approaches targeting NKG2D primarily revolve around CAR technology. Preclinical investigations have illustrated that the utilization of NKG2D-CAR-engineered NK cells, known as NKAE, effectively hindered the progression of tumors in MM models [556]. Clinical findings have shown that the application of NKG2D-CAR-NK cells, created through RNA electroporation, not only reduced the formation of ascites but also led to tumor regression in metastatic lesions among patients with colorectal cancer [605]. Furthermore, the combination of NKG2D-CAR-NK cell therapy with an anti-HER2/NKG2D bispecific antibody exhibited remarkable anti-cancer effectiveness, even in cases of HER2-positive tumors lacking NKG2DL expression [606].

Thus far, numerous clinical trials involving CAR-NK cells have been implemented for various cancer types. In the phase 1/2 clinical study of anti-CD19 CAR-NK therapy for lymphoid tumors, the administration of CAR-NK cells achieved a response rate of 73%, without cytokine release syndrome, neurotoxicity, or GVHD [599]. In order to improve readability, we have compiled a comprehensive list of ongoing or completed clinical trials, excluding those withdrawn or terminated (Table 7). The findings of multiple studies have demonstrated that CAR-NK represents a promising therapeutic approach for both hematological malignancies and solid tumors [607–610]. In conclusion, abundant evidence indicates that NK cell-based therapeutic strategies for tumor treatment occupy a prominent and substantial position in cancer immunotherapy.

**Table 7:** Ongoing or completed clinical trials of CAR-NK therapies

NCT number	CAR target	NK cell source	Targeting tumor	Phase	Status
NCT03056339	CD19	UCB	Hematological malignancies	1/2	Completed
NCT05563545	CD19	Non-referred	ALL	1	Completed
NCT05654038	CD19	HPCs	B-cell lymphoma	1/2	Recruiting
NCT05092451	CD70	UCB	Hematological malignancies	1/2	Recruiting
NCT05703854	CD70	UCB	Solid tumors	1/2	Recruiting
NCT05842707	CD19/CD70	UCB	B-cell NHL	1/2	Recruiting
NCT05410717	Claudin6	PBMCs	Reproductive system tumors	1/2	Recruiting
NCT04847466	PD-L1	NK92	GEJ cancers or HNSCC	2	Recruiting
NCT05472558	CD19	UCB	B-cell NHL	1	Recruiting
NCT04887012	CD19	Non-referred	B-cell NHL	1	Recruiting
NCT05213195	NKG2D	Non-referred	Colorectal cancer	1	Recruiting
NCT05528341	NKG2D	NK92	Solid Tumors	1	Recruiting
NCT05645601	CD19	Non-referred	Hematological Malignancies	1	Recruiting
NCT05008575	CD33	Non-referred	AML	1	Recruiting
NCT05507593	DLL3	NK92	Extensive-stage SCLC	1	Recruiting
NCT05410041	CD19	Non-referred	Hematological Malignancies	1	Recruiting
NCT04623944	NKG2D ligands	Non-referred	Hematological Malignancies	1	Recruiting
NCT05020678	CD19	Non-referred	Hematological Malignancies	1	Recruiting
NCT05667155	CD19/CD70	UCB	B-cell NHL	1	Recruiting
NCT04796675	CD19	UCB	Hematological Malignancies	1	Recruiting
NCT05665075	CD33	iPSC	AML	1	Recruiting
NCT05601466	CD33	iPSC	AML	1	Recruiting
NCT05379647	CD19	iPSC	B-cell Malignancies	1	Recruiting
NCT05182073	BCMA	iPSC	Multiple myeloma	1	Recruiting
NCT05336409	CD19	iPSC	Hematological Malignancies	1	Recruiting
NCT03383978	HER2	NK92	Glioblastoma	1	Recruiting

*UCB* Umbilical cord blood, *ALL* Acute lymphoblastic leukemia, *HPCs* Hematopoietic progenitor cells, *NHL* Non-Hodgkin lymphoma, *PBMCs* Peripheral blood mononuclear cells, *GEJ* Gastroesophageal junction, *AML* Acute myeloid leukemia; SCLC: Small cell lung cancer; iPSC: Induced pluripotent stem cell

## Targeting granulocytes or other innate immune cells for cancer treatment

Granulocytes encompass a diverse group of leukocytes, namely neutrophils, basophils, eosinophils, and mast cells [611]. These cells are part of the innate immune system and, upon activation, release molecules that stimulate the immune response to defend against infections [612]. Besides, granulocytes are implicated in various conditions such as asthma, allergies, autoimmune diseases, and cancers [613]. Among the granulocytes, neutrophils are the most abundant (50–70% of circulating leukocytes in humans), followed by eosinophils [614]. Basophils are the least common, constituting less than 1% of circulating leukocytes [615]. Mast cells, on the other hand, predominantly reside in tissues [616].

### Neutrophil-targeted therapies

Neutrophils play a crucial role as the first line of defense against microbial infections and are also implicated in various inflammatory diseases [617–619]. Recently, there has been growing interest in understanding the versatile roles of neutrophils in cancer initiation and progression [620]. Specifically, tumor-associated neutrophils (TANs) exhibit diverse behaviors influenced by external stimuli from the TME [621]. These TANs can switch antitumor (N1) and protumor (N2) phenotypes [622]. N1 neutrophils could eliminate tumor cells by direct cytotoxic activities and indirectly stimulating adaptive immune responses. Contrarily, N2 neutrophils promote cancer cell proliferation, angiogenesis, and immune evasion [623, 624]. It has been confirmed that IFN-I polarizes neutrophils toward the antitumor N1 phenotype, while TGF-β drives the polarization toward the protumor N2 phenotype [625–629]. The N1/N2 nomenclature for TAN is inspired by the classification of activation states observed in TAM. However, specific surface markers reliably distinguishing between N1 and N2 TANs are unavailable, unlike TAM [630]. The plasticity of neutrophil polarization underscores the dynamic alterations of their functions in the TME. Neutrophils can adopt different activation states and functions depending on the specific cues and signals they encounter [631–633]. Understanding the precise mechanisms and factors that drive neutrophil polarization in the TME is a research hotspot, that provides insights into potential therapeutic strategies by modulating neutrophil functions [628]. Recent studies have suggested that targeting neutrophils could be a potential strategy in cancer therapy, mainly by inhibiting their protumoral capabilities [634, 635].

#### The roles of TANs in cancer development

TAN is generally regarded as a protumor factor in multiple types of cancers [636]. Numerous studies have demonstrated that the high neutrophil-to-lymphocyte ratio correlates with poor outcomes [637–640]. TAN-derived molecules, including ROS, protumor cytokines/chemokines, and enzymes, contribute to cancer initiation, progression, and metastasis [633]. ROS released by neutrophils leads to DNA damage and mutations, which are important to carcinogenesis [641, 642]. Besides, ROS from neutrophils promotes HIF-1α-VEGF axis-mediated angiogenesis and triggers oncogenic pathways in cancer cells such as MAPK, PI3K-AKT, and IKK/NF-κB [643]. Additionally, ROS is associated with immune escape, cancer-related inflammation, EMT, and treatment resistance in multiple types of cancers [643–645]. Apart from ROS, neutrophils secret other protumor cytokines and chemokines such as TGF-β, oncostatin M (IL-6 superfamily member), CCL4, CXCL8, BV8, and HGF to facilitate the malignant properties of cancer cells [646–652]. Moreover, some enzymes in neutrophil granules, including neutrophil elastase (NE), cathepsin G (CG), and MMP8/9, participate in extracellular matrix remodeling, EMT, and activation of oncogenic pathways such as EGFR-MAPK and PI3K-AKT signaling [653–656].

Significantly, extracellular traps (NETs) released by neutrophils have emerged as a pro-tumor factor. On the one hand, NETs assist in tumor growth and distant metastasis by entrapping circulating tumor cells [657–661]. On the other hand, NETs facilitate immune evasion and shield tumor cells from immune cytotoxicity [662]. Research has shown that tumor-produced CXCR1/2 ligands stimulate the generation of NETs, which envelop tumor cells, impeding their interactions with NK cells and CD8^+^ T cells. Consequently, tumor cells wrapped in NETs evade immune attacks. Disrupting NET formation with inhibitors of protein arginine deiminase 4 counteracts NET-mediated immune evasion and synergizes with immune checkpoint inhibitors in mouse models of cancer [663]. Similarly, in pancreatic ductal adenocarcinoma, IL-17 promotes neutrophil recruitment, NET formation, and the exclusion of CD8^+^ T cells. Blocking NET formation through IL-17 inhibition enhances the efficacy of immune checkpoint inhibitors [664].

Although TAN is identified as a risk factor for cancer patients in a majority of studies, TAN plays antitumor roles in certain circumstances. TANs could directly eradicate tumor cells by H_2_O_2_-mediated lethal Ca^2+^ influx, FasL-Fas interaction, and nitric oxide release [665–667]. Besides, TANs enhance the antitumor immune response by triggering ADCC, recruiting and activating T cells, and acquiring antigen presentation capabilities in some subsets [668–670]. TANs support T cell activation and priming not only by secreting proinflammatory factors such as TNF-α and CathG but also by NET-mediated downregulation of T cell activation threshold [671]. Collectively, TANs play complex roles in tumorigenesis and tumor progression. Further research is needed to fully understand the precise contributions and potential therapeutic targeting of TANs in cancer.

#### Manipulating TANs for cancer therapies

Various strategies for targeting TANs have been developed, including inhibiting their recruitment, inhibiting their functions, and reprogramming them toward the antitumor phenotype. These strategies aim to either eliminate or reprogram TANs to exert beneficial effects in cancer therapy (Table 8). Similar to PMN-MDSCs, the recruitment of TANs into the TME is mainly driven by CXCR2/CXCR4 signaling [672–674]. Agents blocking the CXCLs/CXCR2 axis effectively retard tumor progression by abrogating TAN-mediated protumor effects in preclinical models [675–677]. CXCR2 selective antagonists such as Navarixin and SCH527123 decrease neutrophil levels in patients [678–680]. Besides, CXCR4 silence in myeloid cells enhances NK cell-mediated immune surveillance against tumor cells, and systemic CXCR4 antagonist administration effectively suppresses tumor growth in melanoma models [681]. In the phase 2 study of pancreatic ductal adenocarcinoma (NCT02826486), CXCR4 antagonist BL-8040 combined with pembrolizumab and chemotherapy significantly reduces PMN-MDSC/TAN but increases T cell infiltration in the TME [682]. Also, suppressing TAN accumulation by lorlatinib treatment improves anti-PD-1 therapy in murine tumor models [683]. Moreover, tumor-derived oxysterols, the IL-23/IL-17/G-CSF axis, and the complement component 5-a (C5a) are also identified as neutrophil attractants [684–686]. Therefore, therapies blocking oxysterols, G-CSF, and C5a might be promising TAN-targeting strategies in the future [687].

**Table 8:** Tumor-associated neutrophil (TAN)-targeted cancer therapies

Classification	Target	Agents	Representative clinical trials	Cancer types	Phase
Inhibiting TAN recruitment into the TME	CXCR1/2	Reparixin	NCT02370238	TNBC	2
		Navarixin	NCT03473925	Solid tumors	2
		SX-682	NCT04574583	Solid tumors	1/2
	CXCR2	AZD5069	NCT03177187	Prostate cancer	1/2
	CXCL8	HuMax-IL8	NCT02536469	Solid tumors	1
	CXCR4	BL-8040	NCT02826486	Pancreatic adenocarcinoma	2
		BMS-936564	NCT01120457	Leukemia	1
		MSX-122	NCT00591682	Solid tumors	1
		Plerixafor	NCT01236144	Leukemia	1/2
		MB1707	NCT05465590	Solid tumors	1
Increasing the antitumor activity of TANs but undermining their protumor capabilities	PD-1/PD-L1	Nivolumab	NCT02713867	NSCLC	3
		Pembrolizumab	NCT02555657	TNBC	3
		Atezolizumab	NCT03125902	TNBC	3
	CD47/SIRPα	Hu5F9-G4	NCT03922477	Leukemia	1
		TTI-621	NCT02663518	Hematologic and solid malignancies	1
		CC-90002	NCT02641002	Leukemia	1
	COX-2	Celecoxib	NCT03026140	Colon cancer	2
	S100A8/A9	Tasquinimod	NCT01234311	Prostate cancer	3
Reprogramming TAN toward the antitumor phenotype	TGF-β	M7824	NCT03631706	NSCLC	3
		BiTP	NCT05028556	Solid tumors	1
		SHR-1701	NCT05179239	Cervical cancer	3
	NAMPT	ATG-019	NCT04281420	Hematologic and solid malignancies	1

*NSCLC* non-small cell lung cancer, *TNBC* triple-negative breast cancer, *TAN* tumor-associated neutrophil, *TGF-β* transforming growth factor β, *NAMPT* nicotinamide phosphoribosyltransferase, *COX-2* cyclooxygenase-2, *TME* tumor microenvironment

Besides, some therapies increase the antitumor activity of TANs but undermine their protumor capabilities. In murine colon tumor models, PD-L1^+^ TANs dampen the cytotoxic activities of PD1^+^ NK and T cells, leading to cancer immune escape [683, 688, 689]. Blocking the PD-1/PD-L1 axis relieves the immunosuppressive functions of PD-L1^+^ TANs and strengthens the tumor-killing activities of TANs [690]. Parallelly, anti-CD47/SIRPα immunotherapy magnifies TAN-mediated ADCC and inhibits tumor growth [691–693]. Moreover, S100A9^+^ neutrophils propel M2 polarization in a COX-2-dependent manner [694]. Nuclear S100A9 binds to C/EBPβ, which cooperatively activates *Cox-2* promoter and initiates the expression of PGE2, leading to M2 polarization [694]. In patients with advanced solid tumors, COX inhibitor combined with immune checkpoint inhibitor shows superior antitumor activity to immune checkpoint inhibitor monotherapy [695]. Theoretically, strategies targeting S100A9 or COX could prevent TAN-mediated immunosuppression, needing further clinical validations.

As we mentioned above, TGF-β is the core component stimulating TAN polarization toward the protumor N2 phenotype. Hence, neutralizing TGF-β in the TME reprograms the TAN phenotype and promotes immune clearance against tumor cells [629]. At present, several TGF-β blockade therapies have been undergoing clinical evaluation, especially anti-PD-L1/TGF-β bispecific or bifunctional antibodies [696, 697]. In the preclinical and clinical studies, anti-PD-L1/TGF-β bispecific antibodies such as M7824, YM101, and BiTP exhibit potent activities and achieve higher response rates in multiple types of cancers, relative to historical data [698–700]. Besides, nicotinamide phosphoribosyltransferase (NAMPT) contributes to the switch toward N2 TAN, while NAMPT inhibitors impair TAN-mediated tumorigenesis in murine tumor models [701]. In summary, by understanding the intricate interactions between TANs and the TME, novel therapeutic approaches can be developed to harness the antitumor potential of neutrophils while mitigating their protumoral effects. Targeting TANs holds promise for enhancing the efficacy of cancer treatments and improving patient outcomes. Future studies and clinical trials will be instrumental in translating these findings into practical and effective therapeutic strategies for cancer patients.

Additionally, neutrophils have shown the potential as carriers for drug delivery [702]. As the most abundant white blood cells, neutrophils can effectively traverse formidable barriers like the blood–brain barrier, facilitating the transport of drugs or nanoparticles to inflamed tissues such as tumors [703]. Preclinical research has demonstrated that neutrophils loaded with liposomes containing paclitaxel can effectively infiltrate the murine brain and suppress glioma recurrence following tumor resection. Enhanced inflammatory signals in the brain post-surgery promote the release of liposomal paclitaxel from neutrophils, enabling the effective delivery of paclitaxel to the remaining tumors [704]. Besides, Chang and colleagues have devised anti-glioblastoma CAR-neutrophils derived from human pluripotent stem cells, which can load and transport glioblastoma-targeted nanodrugs without necessitating the induction of additional inflammation in tumors, such as that resulting from surgery [705]. Collectively, these systems for drug and particle delivery utilizing neutrophils exhibit potent antitumor activity and a reduced risk of off-target effects, holding significant promise for clinical translation.

### Eosinophil-targeted therapies

Eosinophils are originally believed to play a vital role in parasitic infection and allergic diseases [706, 707]. Although tumor-infiltrating eosinophils (termed tumor-associated tissue eosinophils, TATEs) were observed a century ago, their roles in cancer development are still unclear and controversial [708, 709]. For instance, TATEs are a favorable prognosis predictor for head and neck cancer and colon cancer [710, 711] but a risk factor for Hodgkin’s lymphoma [712]. This controversy could partly be explained by insufficient patient quantity and technical differences, especially staining methods for TATEs [713, 714]. Besides, the heterogeneity and plasticity of the eosinophils also lead to opposing functions in response to diverse stimuli [715, 716].

The mechanisms of TATE recruitment are still not fully understood, which might be mediated by IL-5-CCR3 signaling and chemokines such as eotaxin [717–719]. Once eosinophils infiltrate into the TME, they could exert cytotoxic activities by secreting granule proteins, including major basic protein (MBP), eosinophil-derived neurotoxin, peroxidase, and cationic protein [720]. Besides, the co-culture experiments using eosinophils and colon cancer cells demonstrate that TNF-α and granzyme-A also participate in eosinophil-mediated tumor killing [721]. Further explorations indicate that IL-18 facilitates the antitumor effects of eosinophils by increasing the expression of adhesion molecules [722]. Eosinophils express functional natural killer cell-associated killing receptors such as CD244, and eosinophil activation by CD244 cross-linking induces cytotoxicity against tumor cells [723, 724]. Besides, IL-12 and IL-10 from eosinophils downregulate the migration and enhance the adhesion of tumor cells by increasing their E-cadherin expression [725]. Furthermore, eosinophils could mediate antitumor response in indirect manners. TATEs attract CD8^+^ T cells into the TME by secreting CCL5, CXCL9, and CXCL10 [726]. Additionally, activated TATEs promote macrophage polarization toward the antitumor phenotype [726]. Also, the antitumor properties of TATEs are associated with TATE-orchestrated vasculature normalization [727].

On the contrary, TATEs possess protumor capabilities in some cancer contexts. TATEs increase Treg accumulation by secreting CCL22 and undermine T cell response by generating IDO in the TME [728, 729]. Moreover, thymic stromal lymphopoietin generated by tumor cells could induce TATEs to produce multiple cytokines (e.g., IL-10, IL-4, IL-5, and IL-13), promoting cancer cell proliferation and inducing macrophage polarization toward the protumor M2-like phenotype [730, 731]. Thymic stromal lymphopoietin also promotes TATEs to secret VEGFA, improving tumor angiogenesis [732]. TATE-derived molecules such as EGF, FGF, and PDGF directly support tumor growth [733]. TATEs also accelerate tumor metastasis and metastatic seeding by TGF-β-induced EMT and MMP2/9-mediated matrix remodeling [734, 735].

Eosinophil level has been identified as a potential biomarker for cancer immunotherapies. Increased eosinophil abundance (absolute eosinophil count) is associated with higher response rates and more prolonged survival in patients treated with ipilimumab [736–738]. Besides, eosinophilia is positively correlated to the efficacy of anti-PD-1 treatment in patients with advanced melanoma and Hodgkin’s lymphoma [739–742]. Mechanistically, immune checkpoint inhibitors stimulate CD4^+^ T cells to produce IL-5, promoting systemic eosinophil proliferation [743]. Then, treatment-induced IL-33 improves eosinophil infiltration into the TME and CD8^+^ T cell activity in an eosinophil-dependent manner [743, 744].

While the current understanding of eosinophils in the TME is limited, there is an urgent need to delve into their roles to develop effective strategies for cancer treatment. Due to the heterogeneity and plasticity of the eosinophils in different types of cancers, eosinophil-targeted therapies might need to be carried out individually. For tumors where eosinophils with protumor properties, targeting them becomes an attractive avenue. In this circumstance, eosinophil-depleting agents such as anti-IL-5 and anti-eotaxin antibodies might be an optional strategy. However, targeting eosinophils becomes more complex when they exhibit antitumor activities, as extensive antigen-independent degranulation may result in severe adverse effects. It is crucial to design drugs that selectively target tumor cells while sparing normal cells [708, 727, 745].

### Targeting basophils and mast cells

Basophils and mast cells share certain features, such as the presence of basophilic granules in the cytoplasm, the expression of the high-affinity IgE receptor (FcεRI), and the release of proinflammatory substances like cysteinyl leukotrienes and histamine [746, 747]. These similarities initially led to the mistaken notion that basophils were the circulating counterparts or precursors of tissue-resident mast cells. However, extensive evidence now demonstrates clear disparities between human basophils and mast cells in terms of their morphology, ultrastructure, immunological characteristics, biochemical composition, and pharmacological responses [615]. As a result, the previous concept that basophils serve as the precursor or counterpart to tissue mast cells is no longer accepted [748, 749]. Recent studies demonstrate that these cells not only participate in allergic diseases, chronic or autoimmune inflammation, and defense against infections, but also play a vital role in cancer development [750, 751].

In specific human solid tumors, alterations in the count of circulating basophils are associated with disease progression. Basophilia, an increase in basophil count, is linked to improved prognosis of patients with NSCLC, melanoma, ovarian cancer, and glioblastoma [752–756]. On the contrary, basopenia, a decrease in basophil count, is associated with an unfavorable prognosis of colorectal cancer [757–759]. Indeed, the effects of basophils are diverse in different tumor settings: either in protumor or antitumor roles [760]. Basophils and their mediators may exhibit antitumor effects in specific contexts. Basophil recruitment is facilitated by factors like VEGF and IL-3 released by cancer and immune cells in the TME by VEGFR2 and IL-3Rα pathways [761, 762]. Intratumoral basophils release CCL3 and CCL4, which recruits CD8^+^ T cells to the TME, resulting in tumor regression in murine melanoma models [763]. Tumor-derived IL-33 activates basophils and enhances their ability to kill cancer cells [764, 765]. In ovarian cancer patients, the presence of an activated basophil signature is associated with better outcomes [755].

In contrast, basophils have been identified as protumor factors under certain circumstances. A key player in this process is Galectin-3 (Gal-3), a protein highly expressed by cancer cells and linked to poor prognosis. Gal-3 promotes immunosuppression within the TME [766]. Laboratory studies have demonstrated that Gal-3 on cancer cells can activate basophils, leading to the release of significant amounts of IL-4 and IL-13 [767, 768]. These cytokines, in turn, stimulate the polarization of M2-like TAMs, further undermining antitumor immune response [769]. Besides, IL-4-producing basophils accumulate in tumor-draining lymph nodes, regulating the TME and promoting the protumor Th2 inflammation [770]. Additionally, basophils promote tumor angiopoiesis by secreting VEGF-A [771]. Developing a comprehensive framework of the molecular mechanisms controlled by basophils within the TME may pave the way for the creation of innovative pharmacological and immunological approaches. These strategies could be utilized to regulate basophil activities, potentially impeding cancer development. So far, some basophil-targeted therapies, such as anti-IL-3Rα/CD123 antibodies, show promising activities in hematologic malignancies [762].

Similar to basophil, mast cell is a double-edged sword in cancer development as well [772–774]. Although tumor-infiltrated mast cells were reported a hundred years ago, it is still unclear whether these innate cells contribute to tumor progression or regression [775–779]. Recent studies have demonstrated that mast cells act as a protumor or antitumor factor depending on cancer types, tumor stages, and TME statuses [780]. On the one hand, mast cells exert protumor activity through secreting proangiogenic factors, releasing growth factors, reshaping the extracellular matrix, and suppressing antitumor immune response [616, 781]. Specifically, accumulated mast cells in the TME generate multiple proangiogenic molecules (such as VEGF-A/B, heparin, FGF, histamine, and stem cell factor) and lymphangiogenic cytokines (VEGF-C/D), promoting tumor angiogenesis and metastasis [782–787]. Besides acting as an important source of proangiogenic cytokines, mast cells also participate in cancer immune evasion. Mast cells secret anti-inflammatory cytokines like IL-10 and TGF-β and mobilize Tregs and MDSCs [788, 789]. On the other hand, mast cells possess antitumor properties under certain conditions. They not only induce cytotoxic effects on tumor cells but also attract immune effector cells [772, 790, 791]. Mast cells selectively recruit other immune cells by regulating cell adhesion and vascular permeability and releasing chemokines. CCL3, CCL5, CXCL10, and LTB4 from mast cells guide T-cell infiltration into the TME [792, 793]. Also, mast cells induce the chemotaxis of neutrophils and NK cells by secreting IL-8 [794, 795].

Hereto, manipulating the recruitment, activation, and status of mast cells would be valuable in controlling tumor growth [796, 797]. UV radiation induces the migration of skin mast cells by CXCR4-CXCL12 signaling while interrupting the CXCR4-CXCL12 pathway prevents sunlight-caused skin cancers [798]. Besides, mast cell-stabilizing drugs such as infliximab (anti-TNF antibody) suppress colorectal tumor progression [799]. SCF-c-kit pathway is the core signaling regulating mast cell development, and the c-kit inhibitor imatinib mesylate abrogates the influences of mast cells on tumor progression [800]. However, the role of mast cells changes along with cancer types, tumor stages, and mast cell statuses. Therefore, inhibiting the accumulation or functions of mast cells might not benefit all types of cancers.

### Exploiting other innate immune cells

Recently, the importance of other innate immune cells in tumor progression is beginning to come into focus, especially unconventional T cell subsets γδ T cells, NKT cells, and MAIT cells [801]. Relative to conventional T cells, these innate T cells possess limited or semi-invariant TCR repertoires [802–804]. The unconventional T cells activate, mediate, and regulate antitumor response, becoming promising targets for cancer immunotherapy [805].

Human γδ T cells commonly exert antitumor properties upon activation [806]. Activated γδ T cells directly kill tumor cells by releasing cytolytic granules or expressing ligands of death receptors such as FASL and TRAIL [807–809]. Besides, γδ T cells improve the recruitment and functions of other immune cells, including αβ T cells, B cells, NKs, and antigen-presentation cells [810–815]. However, in some specific conditions, γδ T cells possess protumor activities [816]. For example, γδ T cell-derived IL-17 induces the formation of immunoinhibitory TME, supports angiogenesis, and promotes tumor progression [817–820]. Most clinical studies demonstrate that γδ T cell is a favorable biomarker for the prognosis and treatment response of cancer patients [821–825]. Considering their potent antitumor activity, manageable safety profile, and potential in allogeneic adoptive cell therapy, γδ T cells have become promising candidates for cancer immunotherapy [826]. At present, the development of CAR-γδ T cell, TCRγδ-transduced T cell, and γδ T cell-specific engagers has substantially innovated the blueprint for cancer immunotherapy. Multiple bispecific antibodies, such as TRGV9/CD40, TRGV9/CD1d, TRGV9/CD123, TRGV9/EGFR, and TRGV9/HER-2, exhibit potent activity in preclinical hematological and solid malignancy models [827–831]. Besides, anti-butyrophilin 3A (BTN3A) antibody could activate γ9Vδ2 T cells to eradicate tumor cells, and the preliminary data demonstrate that anti-BTN3A therapy is well-tolerated in patients with advanced solid tumors [832]. Moreover, adoptive cell therapies with expanded γδ T cells, CAR-γδ T cells, and γδTCR-engineered T cells also show encouraging activities in preclinical and clinical studies (Table 9) [833–842]. For instance, allogeneic Vδ1 T cells, genetically engineered to express anti-GPC-3 CAR and soluble IL-15, could effectively sustain self-proliferation and inhibit antitumor activity, representing a promising antitumor agent warranting clinical evaluation [843].

**Table 9:** γδ T cell-based cancer immunotherapies

Classification	Targets/Cells	Agents	Preclinical models or clinical trials
γδ T cell engagers	TRGV9/HER2	(Her2)2xVγ9	Preclinical pancreatic cancer model
	TRGV9/EGFR	LAVA-1223	Preclinical colon cancer model
	TRGV9/CD1d	LAVA-051	Leukemia and MM (NCT04887259)
	TRGV9/CD40	LAVA-1278	Preclinical MM model
	TRGV9/CD123	LAVA-1266	Preclinical AML model
	BTN3A	ICT01	Solid and hematological malignancies (NCT04243499 and NCT05307874)
Expanded γδ T cell transfer	Allogeneic Vγ9Vδ2 T cells	Unnamed product	Lung and liver cancers (NCT03183232 and NCT03183219)
	Allogeneic Vδ1 T (DOT) cells	GDX012	AML (NCT05001451)
	MGMT-modified γδ T cells	INB200	Glioblastoma (NCT04165941)
γδTCR-engineered T cell transfer	Vγ9Vδ2 TCR-engineered αβ T cells	TEG002	MM (NCT04688853)
	αβ T cells with anti-CD19 AbTCR	ET190L1	Lymphoma (NCT03415399)
	αβ T cells with anti-α-fetoprotein AbTCR and glypican-3-targeted co-stimulatory molecule	ET140203	HCC (NCT04502082 and NCT04634357)
CAR-γδ T cells	NKG2DL-targeting CAR-γδ T cells	CTM-N2D	Solid tumors (NCT04107142)
	CD20-targeting CAR-γδ T cells	ADI-001	B Cell Malignancies (NCT04735471 and NCT04911478)
	Glypican-3-targeting CAR-γδ T cells expressing soluble IL-15	ADI-002	Preclinical HCC model
	CD19-targeting CAR-γδ T cells	Unnamed product	Preclinical CD19^+^ leukemia model
	MUC1-Tn-targeting CAR-γδ T cells	Unnamed product	Preclinical gastric cancer model
	CD123-targeting CAR-DOT cells	Unnamed product	Preclinical AML model

*MM* multiple myeloma, *AML* acute myeloid leukemia, *MGMT* methylguanine DNA methyltransferase, *HCC* hepatocellular carcinoma, *TCR* T cell receptor, *DOT* Delta One T, *TCR* T cell receptor, *CAR* chimeric antigen receptor

MAIT cells are a cluster of evolutionarily conserved unconventional T cells, with enormous potential in cancer immunotherapy [844]. MAIT cells could kill tumor cells by MHC-related molecule 1 (MR1)-TCR or NK cell-activating receptors [845, 846]. Apart from direct antitumor activity after activation, some basic studies indicate that MAIT cells also possess immunomodulatory functions, especially enhancing the functions of NK cells [847]. Also, accumulated MAIT cells are closely associated with improved response to anti-PD-1 treatment [848–850]. Due to their potent antitumor ability, high safety profile, and the ability to undergo genetic modification, MAIT and CAR-MAIT cells have emerged as encouraging options for cancer immunotherapy [844]. The high abundance of MAIT cells in the gastrointestinal tract, lung, and cervix suggests that cancers originating in these mucosal-associated peripheral tissues might be more likely to benefit from MAIT cell-based treatment [844].

NKT cells are a group of unconventional T cells that recognize glycolipids presented by CD1d [851]. Type I NKT cells, also termed invariant natural killer T cells (iNKTs), express invariant TCR α chain (Vα24-Jα18), with antigen specificity to synthetic glycolipid alpha-galactosylceramide (αGalCer) [852]. On the contrary, type II NKT cells have diverse TCR repertoires with poorly defined antigen specificity [853]. Commonly, most NKT-involved immunotherapies are based on iNKT cells [854]. Upregulated functions or levels of iNKTs are positively correlated with improved outcomes in lung cancer, colon cancer, neuroblastoma, and multiple myeloma [855–858]. iNKT cells could directly kill CD1d^+^ cancer cells [859–864]. Besides, iNKT cells boost antitumor response by regulating other immune cells. For example, tumor-infiltrating iNKT cells induce the polarization of CD1d^+^ TAMs toward antitumor M1-like or directly deplete them [865, 866]. Moreover, iNKT cells promote the maturation of DCs and convert MDSC to immunostimulatory antigen-presentation cells [867–869]. At present, unmodified and engineered NKT therapies have been developed for cancer immunotherapy (Table 10). Redirected NKT therapies endow NKT cells with cancer specificity and antitumor capability by CARs, cancer-specific TCRs, and anti-CD1d antibody fusion proteins [854]. NKT cells expressing CARs recognizing cancer-associated antigens exhibit potent activity in several murine tumor models [870–875]. In the phase 1 clinical study of neuroblastoma (NCT03294954), anti-GD2 CAR-NKT cells achieve encouraging efficacy with a tolerable safety profile [876]. Moreover, the efficacies of TCR-modified NKT and anti-CD1d antibody fusion proteins have been validated in a series of preclinical tumor models, needing further validation in clinical studies [877–881]. Generally, the powerful antitumor properties of unconventional T cells have been well-accepted, and regulating these components might provide an effective immunoprotection against cancers [882].

**Table 10:** Representative clinical trials of CAR-NKT therapies

Clinical trials	Agents	Cancer types	Phase	Status
NCT03294954	Anti-GD2 CAR and IL-15 expressing NKTs	Neuroblastoma	1	Active, not recruiting
NCT03774654	Anti-CD19 CAR and IL-15 expressing NKTs	B cell malignancies	1	Recruiting
NCT05487651	Anti-CD19 CAR and IL-15 expressing NKTs	B cell malignancies	1	Recruiting
NCT04814004	Anti-CD19 CAR and IL-15 expressing NKTs	B cell malignancies	1	Recruiting
NCT02439788	Anti-GD2 CAR expressing NKTs	Neuroblastoma	1	Withdrawn

*CAR* chimeric antigen receptor

## Perspective and conclusion

Immunotherapies have revolutionized cancer treatment, offering promising outcomes and prolonged survival for patients across various cancer types. Current immunomodulatory strategies predominantly focus on harnessing adaptive immunity, utilizing approaches such as immune checkpoint blockade and CAR-T cell therapy. While these approaches have shown remarkable success in some cases, the overall response rates remain limited, highlighting the need for novel therapeutic avenues. In recent years, accumulating evidence has emphasized the crucial role of the innate immune system in orchestrating antitumor immune responses. By recognizing and eliminating cancer cells, as well as modulating adaptive immunity, innate immune cells present a fertile ground for innovative immunotherapeutic interventions.

**Fig. 6:**
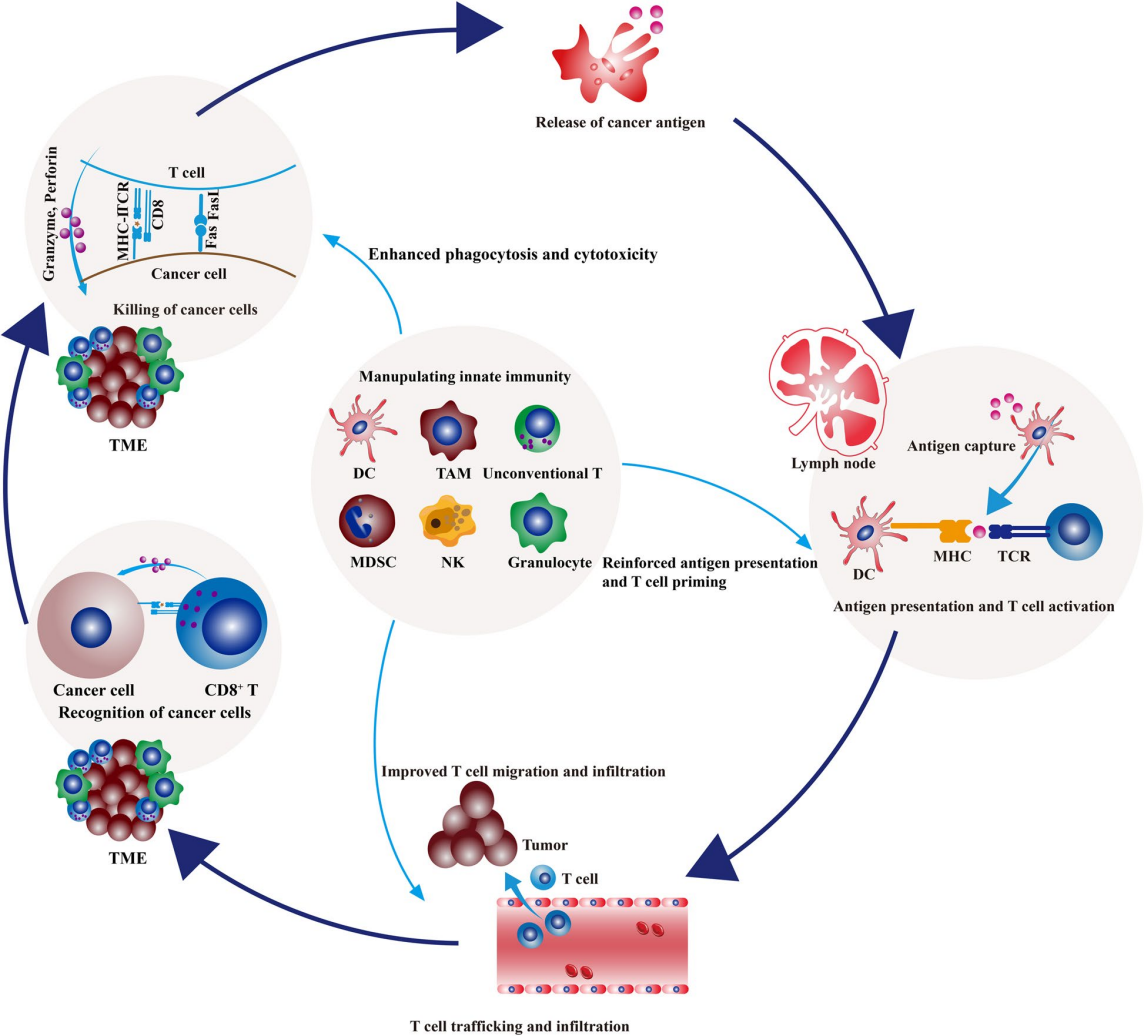
Harnessing innate immunity to improve antitumor immune response. The involvement of innate immunity is crucial for initiating and sustaining adaptive immunity, and it plays a significant role in the overall cancer-immunity cycle. When a tumor is detected, innate immune cells are activated, leading to the enhancement of their effector functions and the destruction of tumor cells. Apart from directly killing tumor cells, innate immune cells participate in priming, expanding, and infiltrating tumor-specific T-cells. Manipulating innate immunity by therapeutic strategies could effectively stimulate antitumor immune response and overcome immune evasion. Abbreviations: DC, dendritic cell; TAM, tumor-associated macrophage; NK cell, natural killer cell; MDSC, myeloid-derived suppressor cell; TME, tumor microenvironment; TCR, T cell receptor; MHC, major histocompatibility complex

Beyond their well-established roles in immune surveillance and clearance of pathogens, innate immune cells actively participate in cancer immune evasion and surveillance. Macrophages, DCs, MDSCs, neutrophils, and NK cells are key components of the innate immune arm that influence the TME and shape antitumor immune responses (Fig. 6). Understanding the intricate interplay between innate immune cells and tumor progression is crucial for developing effective therapeutic interventions. Moreover, exploiting the potential of innate immunity opens new avenues for cancer immunotherapy. Several strategies have emerged that focus on modulating innate immune cells to enhance antitumor responses. STING agonists have shown promising preclinical results by enhancing antitumor immunity and triggering the production of IFN-I. Another promising avenue is the genetically engineered innate cells, such as CAR-macrophages or CAR-NK cells, which have demonstrated potent antitumor activities in preclinical models. Additionally, TLR agonists have been explored to induce the maturation of antigen-presenting cells, augmenting their ability to present tumor antigens to T cells and promote antitumor responses.

Recognizing the interconnectedness of innate and adaptive immunity, combination therapies that simultaneously target both arms of the immune system hold great promise. Immune checkpoint blockade, a mainstay of current immunotherapies, primarily focuses on reversing T cell exhaustion and reinvigorating adaptive immune responses. However, the effectiveness of immune checkpoint inhibitors can be enhanced by incorporating strategies that activate innate immune cells. For instance, combining immune checkpoint blockade with STING agonists can amplify both innate and adaptive immune responses, resulting in synergistic antitumor effects [62]. Similarly, STING agonists can improve CAR-T cell trafficking and persistence in the TME, effectively enhancing the efficacy of CAR-T cells in solid tumors [883–885].

While harnessing innate immunity presents exciting opportunities, several challenges need to be addressed to fully unleash its potential. A comprehensive understanding of the intricate crosstalk between innate immune cells and the TME is crucial for designing effective therapies. Furthermore, strategies targeting innate immunity should carefully consider potential off-target effects and avoid excessive systemic inflammation. Developing robust biomarkers to predict patient response to innate immune-based therapies and selecting optimal combination regimens are additional challenges that warrant attention.

In conclusion, the advent of cancer immunotherapies has revolutionized cancer treatment, but the full potential of the immune system in eradicating tumors is yet to be realized. Exploiting the power of innate immunity offers a promising approach to overcoming current limitations. Innate immune cells play multifaceted roles in modulating antitumor immune responses and can be harnessed through various approaches, including but not limited to STING agonists, CAR-macrophage or -NK cell therapies, metabolic regulators, and innate immune checkpoint blockade. Synergistic combination therapies that simultaneously activate innate and adaptive immunity hold great promise for future advancements in cancer immunotherapy. By expanding our focus beyond adaptive immunity and embracing the potential of the innate immune system, we can develop more effective and personalized treatments for cancer patients. Unrevealing the multifaceted contributions of innate immune cells and exploring their therapeutic potential will propel the field of cancer immunotherapy forward.

## Data Availability

Not applicable.

## Abbreviations

2-DG: 2-Deoxy-D-glucose
αLP: α-Lymphoid precursor
αGalCer: Alpha-galactosylceramide
ADCC: Antibody-dependent cell cytotoxicity
ADCP: Antibody-dependent cell phagocytosis
ARG1: Arginase 1
ATP: Adenosine triphosphate
ATRA: All-trans retinoic acid
Breg: Regulatory B
BM: Bone marrow
BTN3A: Butyrophilin 3A
C5a: Complement 5a
CAF: Cancer-associated fibroblast
CAR: Chimeric antigen receptor
CRT: Calreticulin
cDC: Conventional DC
CDN: Cyclic dinucleotide
cGAMP: Cyclic GMP-AMP
CSF1: Colony-stimulating factor-1
CTLA-4: Cytotoxic T lymphocyte-associated protein 4
CTX: Cyclophosphamide
DC: Dendritic cell
DAMP: Damage-associated molecular pattern
ECM: Extracellular matrix
EGF: Epidermal growth factor
EMT: Epithelial-mesenchymal transition
Eomes: Eomesodermin
FAO: Fatty acid oxidation
FcR: Fc receptor
FcRγ: Fc receptor
FGF: Fibroblast growth factor
Gal-3: Galectin-3
GM-CSF: Granulocyte macrophage-colony stimulating factor
HDACi: Histone deacetylase inhibitor
HMGB1: High-mobility group box 1
HPC: Hematopoietic progenitor cell
ICD: Immunogenic cell death
IDO1: Indoleamine2,3-dioxygenase1
IgG1: Immunoglobulin G1
IKK-ε: Inhibitor of kB kinase ε
ILC: Innate lymphoid cell
iNOS: Inducible nitric oxide synthase
iNKT: Invariant natural killer T cell
iPSC: Induced pluripotent stem cell
ITAM: Immunoreceptor tyrosine-based activation motif
LILRB: Leukocyte immunoglobulin-like receptor B
MAIT: Mucosa-associated invariant T
MBP: Major basic protein
MDSC: Myeloid-derived suppressor cell
MR1: MHC-related molecule 1
M-MDSC: Monocytic MDSC
MMP: Matrix metalloproteinase
Megf10: Multiple epidermal growth factor-like domains protein 10
MoDC: Monocyte-derived DC
NAMPT: Nicotinamide phosphoribosyltransferase
NCAM: Neural cell adhesion molecule
NET: Neutrophil extracellular trap
NK: Natural killer
NKT: Natural killer T
NSCLC: Non-small cell lung cancer
ODN: Oligodeoxynucleotides
PBMC: Peripheral blood mononuclear cell
PD-1: Programmed cell death protein 1
PD-L1: Programmed cell death ligand 1
pDC: Plasmacytoid DC
PDE5: Phosphodiesterase 5
PGE2: Prostaglandin E2
PMN-MDSC: Polymorphonuclear MDSC
PRR: Pattern-recognition receptor
ROS: Reactive oxygen species
TAM: Tumor-associated macrophage
TAN: Tumor-associated neutrophil
TCR: T cell receptor
tdLN: Tumor-associated draining lymph node
TRAIL: TNF-related apoptosis-induced ligand
Siglec: Sialic acid-binding immunoglobulin-like lectin
SIRPα: Signal regulatory protein-α
SFR: SLAM family receptor
TAN: Tumor-associated neutrophil
TATE: Tumor-associated tissue eosinophil
TIM-3: T-cell immunoglobulin and mucin domain 3
TLR: Toll-like receptor
TME: Tumor microenvironment
Treg: Regulatory T
UCB: Umbilical cord blood
VEGF: Vascular endothelial-derived growth factor
